# Nonstructural Protein A238L of the African Swine Fever Virus (ASFV) Enhances Antiviral Immune Responses by Activating the TBK1-IRF3 Pathway

**DOI:** 10.3390/vetsci11060252

**Published:** 2024-06-04

**Authors:** Wei Liu, Lanlan Yang, Chuanyuan Di, Jing Sun, Penggang Liu, Huisheng Liu

**Affiliations:** 1 Institute of Comparative Medicine, College of Veterinary Medicine, Yangzhou University, Yangzhou 225009, China; liuw@yzu.edu.cn (W.L.);; 2 Jiangsu Co-Innovation Center for Prevention and Control of Important Animal Infectious Diseases and Zoonosis, Yangzhou University, Yangzhou 225009, China; 3 State Key Laboratory for Animal Disease Control and Prevention, Lanzhou Veterinary Research Institute, College of Veterinary Medicine, Chinese Academy of Agricultural Sciences, Lanzhou University, Lanzhou 730000, China

**Keywords:** African swine fever virus, A238L, TBK1, IRF3, NF-κB

## Abstract

**Simple Summary:**

A238L, a non-structural protein of the African swine fever virus (ASFV), inhibits the activation of NF-κB by suppressing the HAT activity of p300. Whether A238L also affects the transcriptional activity of IRF3 remains unexplored. Here we first confirmed the ability of A238L to suppress NF-κB-activity in L929 cells. In contrast, A238L did not inhibit but rather increased TBK1 and IRF3 phosphorylation and enhanced innate antiviral immunity in the absence or presence of poly d (A:T) or poly (I:C) stimulation, or herpes simplex virus type 1 (HSV-1) or Sendai virus (SeV) infection. This study reveals an unrecognized role for A238L in promoting antiviral immune responses by activating the TBK1-IRF3 pathway.

**Abstract:**

African swine fever virus (ASFV) is a double-stranded DNA virus with an envelope. ASFV has almost the largest genome among all DNA viruses, and its mechanisms of immune evasion are complex. Better understanding of the molecular mechanisms of ASFV genes will improve vaccine design. A238L, a nonstructural protein of ASFV, inhibits NF-κB activation by suppressing the HAT activity of p300. Whether A238L also affects the transcriptional activity of IRF3 remains unexplored. Here we first confirmed the ability of A238L to suppress NF-κB-activity in L929 cells. A238L inhibits the expression of proinflammatory cytokine genes. In contrast, A238L increased the phosphorylation levels of TBK1 and IRF3 in three different cell lines. A238L increases the IRF3-driven promoter activity and induces IRF3 nuclear translocation. Furthermore, A238L enhanced innate antiviral immunity in the absence or presence of poly d (A:T) or poly (I:C) stimulation, or herpes simplex virus type 1 (HSV-1) or Sendai virus (SeV) infection. This study reveals a previously unrecognized role of A238L in promoting antiviral immune responses by TBK1-IRF3 pathway activation.

## 1. Introduction

African swine fever (ASF) is an acute contagious disease of domestic swine and wild boar [1,2,3]. ASF has spread from Africa to Europe, and recently to China as well as Southeast Asian countries. ASF is a major threat to the global swine industry and food security [4]. ASF virus (ASFV), a double-stranded DNA virus, belongs to the genus Asfivirus [5]. The pathological change of ASFV infections includes widespread hemorrhages in lymphoid tissues and depletion of macrophages and T and B lymphocytes. The ASFV genome (170–193 kb) harbors approximately 150–167 open reading frames [5]. These open reading frames encode many structural and nonstructural proteins, many of which are capable of interfering with the immune response of host cells [6,7,8]. Because of insufficient knowledge of the functions of many ASFV genes, developing an effective vaccine for ASF has met with great challenges. Characterization of the ASFV genes involved in regulating antiviral immunity may help in designing novel strategies for ASF vaccines.

Pattern recognition receptors (PRRs) play important roles in the inflammatory response and innate immunity. These receptors include Toll-like receptors (TLRs), Nod-like receptors (NLRs), RIG-I-like receptors (RLRs), and cytosolic DNA sensors. TLR3 and RLRs sense extracellular and intracellular viral RNA, respectively. RIG-I primarily recognizes viral double-stranded RNA (dsRNA) and short dsRNA from RNA viruses. MDA5 preferentially binds to long dsRNA derived from encephalomyocarditis virus (EMCV). Upon RNA binding to RIG-1 or MDA5, MAVS is recruited and forms an aggregate with a prion-like filament structure in mitochondria. This aggregate functions as a platform for the activation of TANK-binding kinase 1 (TBK1) and IRF3 [9]. Cyclic GMP-AMP synthase (cGAS) senses cytosolic DNA and produces cyclic GMP-AMP (cGAMP) [10]. cGAMP binding to a stimulator of interferon gene-encoded protein (STING), a transmembrane protein in endoplasmic reticulum and mitochondria, triggers STING aggregation and recruitment of TBK1 and other two adaptor proteins, TAB1 and TAB2/3 [11]. The TBK1 complex is relocated to the perinuclear space where it recruits and phosphorylates interferon regulatory gene 3 (IRF3) [12]. Phosphorylated IRF3 forms a dimer and is translocated to the nucleus, then IRF3 induces transcription of type I interferon genes as a transcription factor [13]. ASFV has a long double-stranded DNA (dsDNA) genome and can readily activate the cGAS-STING pathway. However, many nonstructural proteins encoded by the ASFV genome suppress the cGAS-STING pathway by blocking TBK1 activation.

A238L contains several ankyrin repeats homologous to IκBα and can bind to NF-κB and inhibit its transcriptional activity [14,15]. Inhibition of the transcriptional activity of NF-κB downregulates the expression of inflammatory genes such as TNF-α, iNOS, and IL-6 [16,17]. A238L-deficient ASFV produces higher levels of TNF-α in vitro in porcine macrophages and in vivo in pigs than its parental virus [18]. ASFV deficient of A238L and CD2v (EP402R), a glycoprotein that is homologous to the host adhesion molecule CD2 of T and NK cells, induces both humoral and cellular immune responses that partially protect against challenges with the virulent wild-type ASFV virus [19]. Whether A238L regulates IRF3-mediated antiviral immunity remains unknown. Here we report that A238L did not inhibit but rather enhanced the activation of the TBK1-IRF3 axis and antiviral innate immunity. Our study reveals a previously unrecognized role of A238L in stimulating the antiviral immune response.

## 2. Materials and Methods

### 2.1. Reagents

Poly d (A:T) (Cat# tlrl-patn) and poly (I:C) (Cat# tlrl-pic) were obtained from InvivoGen (Shanghai, China). Antibodies against Sp1 (Cat# SC-59) and GAPDH (s Cat# c-47724) were obtained from Santa Cruz Biotechnology, Inc. (Shanghai, China). Antibodies against phosphorylated TBK1 (Ser172) (Cat# 5483), IRF3 (Ser396) (Cat# 4947), TBK1 (Cat# 3504), IRF3 (Cat# 4302), FLAG (DYKDDDDK Tag) (9A3) (Cat# 8146), GFP (Cat# 2955), rabbit IgG (Cat# 7074), and mouse IgG (Cat# 7076) were obtained from Cell Signaling Technology (Danvers, MA, USA). The A238L gene cloned in the pcDNA3.1-3 × flag vector was reported [20]. Alex488-conjugated anti-rabbit IgG (Cat# IC1051G) and Alex594-conjugated anti-mouse IgG (Cat# IC002T) were obtained from R&D Systems (Minneapolis, MN, USA).

### 2.2. Viruses

The GFP-tagged HSV-1 (GFP is fused to the VP26 protein) was a gift from Zengfan Jiang (Peking University, Beijing, China). The GPF-tagged Sendai virus was a gift from Feng Ma (Suzhou Institute of Systems Medicine, Suzhou, China). HSV-1 was propagated and replicated in Vero cells. By inoculating 10-day-old embryonic chicken eggs free of specific pathogens, the Sendai virus was prepared. The titer of Sendai virus was determined by serial dilution by 10 times (10^1^ to 10^9^) and at each dilution (10^5^ to 10^9^) in Vero cells. The standard Reed and Muench method was used to determine the 50% tissue culture infection dose (TCID_50_/100 μL).

### 2.3. Cells

L929 (Cat #CCL-1) and 3D4/21 (Cat #CRL-2843) cells were obtained from the American Tissue Culture Collection (Manassas, VA, USA). L929 cells were cultured in DMEM (Cat # 11965092) supplemented with 10% fetal bovine serum (FBS) (Cat # 10099158). The IPEC-DQ cells are a subclone of the IPEC-J2 cells, which are a porcine intestinal epithelial cell line. IPEC-DQ cells was obtained from Dr. Dongwan Yoo [20]. IPEC-DQ and 3D4/21 cells were cultured in RPMI-1640 (Cat# 11875093) containing 10% FBS.

### 2.4. RT-PCR Analyses

L929 cells were seeded in 12-well plates and transfected with pcDNA3.1 (2 μg) and A238L (2 μg). After a 36 h transfection, the cells were transfected with poly d (A:T) or poly (I:C) (1 μg/mL of each) for another 12 h. The iScript cDNA synthesis kit (Bio-Rad, Hercules, CA, USA) was used for reverse transcription. Quantitative PCR was performed in triplicate using TB Green^®^ Premix Ex TaqTM II (Takara, Dalian, China). The primer sequences are shown in Table 1. The relative mRNA levels were normalized to the β-actin mRNA level according to the ΔΔCT.

### 2.5. Immunoblotting

The L929, IPEC-DQ, and 3D4/21 cells seeded in 12-well plates were incubated with pcDNA (2 μg) and A238L (1, 2, 4 μg) and incubated for 48 h. Alternatively, L929 cells transfected with pcDNA or A238L at 36 h post-transfection were re-transfected with poly d (A:T) or poly (I:C) or infected HSV-1 (1 MOI) or Sendai virus (1 MOI) and then incubated for 12 h. The cells were collected and lysed in NP-40 lysis buffer [20]. Cytosolic and nuclear fractions were separated by using a cell lysate extraction kit (Beyotime Biotechnology, Nanjing, China). Specific primary antibodies were used to detect the proteins of interest, followed by second antibodies and SuperSignal^®^ Western Pico Chemiluminescent Substrate (Pierce Chemical Co., Rockford, IL, USA).

### 2.6. Luciferase Assay

The L929 cells were transfected with the IRF3 or IFN-β promoter-driven luciferase reporter gene minus or plus pcDNA3.1 or A238L using TurboFect Transfection Reagent (Thermo Fisher Scientific Inc., Waltham, MA, USA) according to the manufacturer’s instructions. The Renilla luciferase reporter driven by the β-actin promoter was included as an internal control. After transfection for 48 h, the cells were collected to analyze the luciferase activity using the luciferin substrate, and the results were read on a TECAN plate reader (Phenix Research Products, Hayward, CA, USA). β-actin promoter-driven Renilla luciferase control was used to normalize the relatively light units in each sample. Means ± standard deviations (SD) of triplicate data from one experiment are presented. The experiments were performed at least twice.

### 2.7. Immunofluorescence Staining

The L929 cells seeded on coverslips in 12-well plates were transfected with pcDNA3.1 or A238L (2 μg each) and incubated for 36 h or transfected with poly d (A:T) (1 μg/mL) or poly (I:C) (1 μg/mL) and incubated for 12 h. The cells were fixed and permeabilized with cold 100% methanol with 0.1% Triton X-100 for 20 min and rinsed with PBS. The coverslips were blocked with 5% BSA at room temperature for 1 h and then probed with anti-phosphorylated IRF3 and anti-Flag antibodies (1:100) overnight at 4 °C. Then, the cells were stained with Alex488 anti-rabbit IgG and Alex594 anti-mouse IgG (1:100) at room temperature for 1 h. The cells were then stained with 10 μM DAPI for 5 min. Fluorescent images were observed under a Leica SP8 confocal laser scanning microscope. The percentage of the cells with the pIRF3-positive nucleus in eight randomly selected fields among all cells was calculated. The experiment was repeated twice with similar results.

### 2.8. Flow Cytometry

The L929 cells were incubated with pcDNA (2 μg) or A238L plasmid DNA (2 μg). After transfection for 36 h, the cells were re-transfected with poly d (A:T). The conditioned media were harvested and added to L929 cells infected with GFP-Sendai virus (0.1 MOI) and then incubated for another 12 h. After treatment, the cells were collected to detect the GFP-positive cells using a Beckman Coulter flow cytometer (Model CyAn ADP). The results were analyzed by the FlowJo 8 software. Statistical analysis was performed by calculating the percentage of GFP-positive cells from three independent experiments.

### 2.9. Statistical Analysis

The mRNA levels, mean channel fluorescence index, luciferase activity, IRF3 nuclear staining, and Western blot band densities were statistically analyzed by the unpaired Student’s *t*-test. Statistically significant was defined as a *p*-value of <0.05.

## 3. Results

### 3.1. A238L Inhibits the Expression of Proinflammatory Cytokine Genes

**Figure 1:**
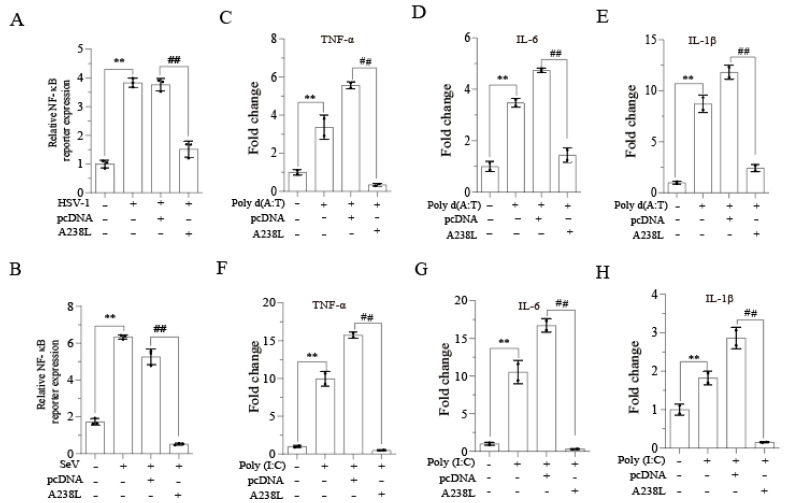
A238L inhibits NF-κB activity and inflammatory cytokine mRNA expression. (**A**,**B**) The NF-κB promoter-driven luciferase reporter gene was transfected into L929 cells. After incubation for 24 h, the cells were infected with HSV-1 or SeV (1 MOI). After 12 h of infection, the cells were collected to analyze the luciferase activity. (**C**–**H**) L929 cells were transfected with pcDNA (2 μg) or A238L (2 μg). After 36 h of transfection, the cells were then mock-transfected or transfected with poly d (A:T) (1μg/mL) or (**C**–**E**) poly (I:C) (1 μg/mL) and (**F**–**H**) incubated for 12 h. Total RNA was extracted. TNF-α, IL-6, and IL-1β gene levels were analyzed by RT-PCR. The results are the means ± SD of three independent experiments. **^,^ ## *p* < 0.01.

Several prior studies have unveiled the ability of A238L to inhibit the NF-κB-mediated transcription of inflammatory cytokine gene expression [14]. We first verified the ability of A238L to suppress NF-κB promoter-driven luciferase reporter gene expression in L929 cells, a murine fibroblastoma cell line that produces abundant IFNs and inflammatory cytokines. As shown in Figure 1A, HSV-1 (Figure 1A) and SeV (Figure 1B) dramatically increased the NF-κB-driven promoter activity, which was blocked by transfection with the A238L expression vector. NF-κB plays a critical role in transcribing three inflammatory cytokine genes, TNF-α, IL-6, and IL-1β [21]. RT-PCR analysis revealed that transfection of poly d (A:T) (Figure 1C–E) and poly (I:C) (F–H) dramatically increased TNF-α, IL-6, and IL-1β mRNA levels in L929 cells, which were further enhanced by transfection with the pcDNA3.1 vector but blocked by A238L.

### 3.2. A238L Enhances the Antiviral Immune Response

**Figure 2:**
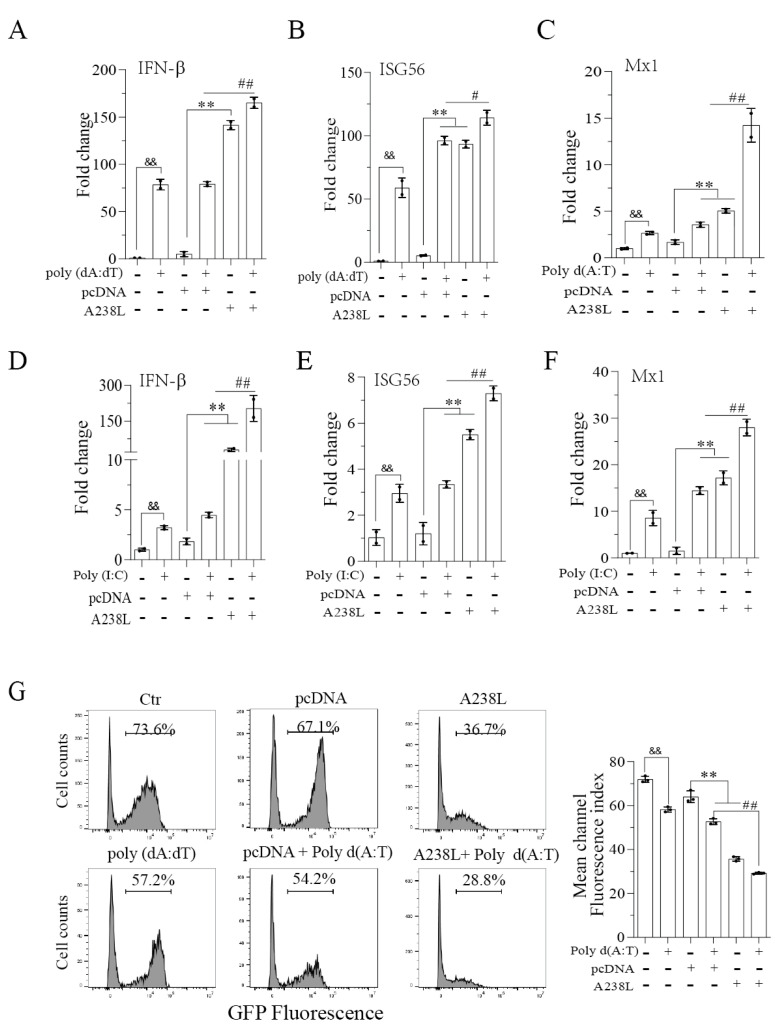
A238L promotes antiviral immunity. L929 cells were transfected with pcDNA (2 μg) or A238L (2 μg). After 36 h of transfection, the cells were mock-transfected or transfected with poly d (A:T) (1 μg/mL) or (**A**–**C**) poly (I:C) (1 μg/mL) and (**D**–**F**) incubated for 12 h. Total RNA was extracted and quantified. Real-time RT-PCR analysis was performed for the IFN-β, ISG56, and MX1 genes. (**G**) L929 cells were transfected with pcDNA, A238L, and poly (dA:dT) alone or in combination. After 48 h of transfection, the conditioned media were collected, diluted 1:10, and added to the L929 cells infected with SeV-GFP (0.1 MOI). Virus replication was analyzed by flow cytometry. The results are the means ± SD of three independent experiments. # *p* < 0.05; ^&&^, **, ## *p* < 0.01.

Here we tested if A238L also interfered with IRF3-mediated gene transcription. As shown in Figure 2A–F, poly d (A:T) or poly (I:C) increased the mRNAs levels of IFN-β, ISG56, and MX1 significantly. pcDNA3.1A transfection alone also weakly or modestly increased the expression of these mRNAs, probably due to the stimulation of cGAS-STING by plasmid DNA. Surprisingly, the mRNA levels of IFN-β, ISG56, and MX1 were much higher in the L929 cells transfected with A238L than those transfected with the pcDNA vector. A238L, in combination with poly d (A:T) (Figure 1A–C) or poly (I:C) (Figure 2D,E), further increased the levels of IFN-β, ISG56, and MX1 mRNAs compared to those transfected with A238L, poly d (A:T), or poly (I:C) alone. To determine whether A238L indeed possesses antiviral activity, the L929 cells infected with GFP-tagged SeV were incubated in the presence of conditioned media from untransfected L929 cells or L929 cells transfected with A238L or poly d (A:T) alone or in combination. As shown in Figure 2G, the conditioned media collected from the L929 cells transfected with A238L or poly d (A:T) significantly decreased GFP fluorescence intensity. The conditioned media collected from L929 cells transfected with A238L plus poly d (A:T) further decreased the GFP-SeV fluorescence intensity, compared to that transfected with pcDNA3.1 plus poly d (A:T) (Figure 2G).

### 3.3. A238L Increases the IRF3-Driven Promoter Activity

**Figure 3:**
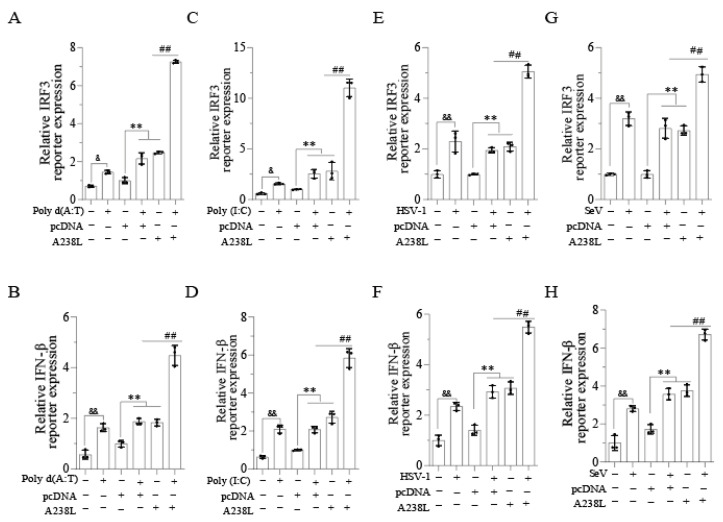
A238L enhances the IRF3- and IFN-β-promoter-driven activity. (**A**) L929 cells were transfected with the IRF3- (**A**–**G**) or IFN-β-promoter (**B**–**H**)-driven luciferase reporter gene plus A238L or pcDNA3.1 as a control. After 36 h of incubation, the cells were transfected with poly d (A:T) or poly (I:C) or infected with HSV-1 or SeV (1 MOI). After 12 h of incubation, the cells were collected to analyze the luciferase activity. The results are the means ± SD of three independent experiments. ^&^ *p* < 0.05, ^&&^, **, ## *p* < 0.01.

IRF3 plays a crucial role in inducing IFN-I and ISG expression [22]. We next explored the effect of A238L on IRF3- and IFN-β-promoter-driven luciferase gene expression. As shown in Figure 3A–D, A238L, poly d (A:T), or poly (I:C) alone significantly increased the IRF3- and IFN-β-promoter-driven luciferase expression. A238L in combination with poly d (A:T) (Figure 3A,B) or poly (I:C) (Figure 3C,D) further increased the IRF3- and IFN-β-promoter-driven luciferase expression, compared to that transfected with poly d (A:T) or poly (I:C) alone. HSV-1 or SeV infection alone increased the IRF3- and IFN-β-promoter-driven luciferase expression significantly (Figure 3E–H). A238L also enhanced the HSV-1- or SeV-induced IRF3- and IFN-β-promoter-driven luciferase expression (Figure 3E–H). These observations collectively suggest that A238L enhances ISG expression by activating IRF3.

### 3.4. A238L Enhances TBK1 and IRF3 Phosphorylation

**Figure 4:**
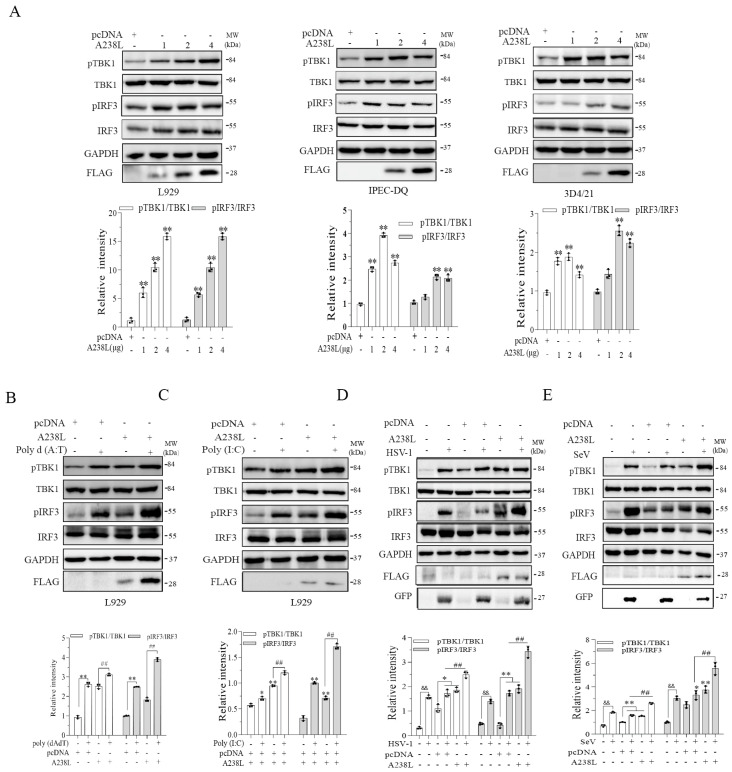
A238L enhances TBK1 and IRF3 phosphorylation. (**A**) L929, IPEC-DQ, and 3D4/21 cells were transfected with pcDNA (2 μg) or different amounts of A238L plasmids. After incubation for 48 h, the cells were collected to analyze the phosphorylation of TBK1 and IRF3 and then re-probed for total proteins by Western blot. GAPDH and FLAG were detected as loading control and transfection control, respectively. ** *p* < 0.01, compared to the pcDNA3.1 control. (**B**–**E**) L929 cells were transfected with pcDNA(2 μg) or A238L plasmid DNA (2 μg). After 36 h incubation, the cells were transfected with poly d (A:T) (**B**) or poly (I:C) (**C**) or infected with HSV-1 or Sendai virus (0.1 MOI) and incubated for 12 h. The cells were collected to analyze the phosphorylation of TBK1 and IRF3 and then re-probed for total proteins by Western blot. GAPDH and FLAG were detected as loading control and transfection control, respectively. Relative phosphorylation levels were semi-quantified using Image J (v1.8.0.345) software. The results are presented as bar graphs. The results are the means ± SD of three independent experiments. * *p* < 0.05; ^&&,^ **, ## *p* < 0.01.

Cytosolic DNA and RNA activate the TBK1-IRF3 pathway through their sensors and induce the expression of type I interferons [10,23]. We next investigated the effect of A238L on TBK1 and IRF3 phosphorylation. As shown in Figure 4A, A238L increased TBK1 and IRF3 phosphorylation in a dose-dependent manner in L929 cells and two cell lines of swine origin: IPEC-DQ, a porcine intestinal epithelial cell line, and 3D4/21, a porcine macrophage cell line. The levels of A238L expression were also dose-dependently elevated in these cells transfected with increasing amounts of A238L plasmid DNA (Figure 4A and Figures S1–S6). Poly d (A:T) or A238L alone significantly induced TBK1 and IRF3 phosphorylation (Figure 4B). Poly d (A:T) plus A238L further increased TBK1 and IRF3 phosphorylation, compared to that transfected with poly d (A:T) and pcDNA or A238L alone. Similar observations were made with poly (I:C) (Figure 4C and Figures S1–S6). Consistently, A238L enhanced HSV-1- or SeV-induced TBK1 and IRF3 phosphorylation in L929 cells (Figure 4D,E and Figures S1–S6 and S8).

### 3.5. A238L Induces IRF3 Nuclear Translocation

**Figure 5:**
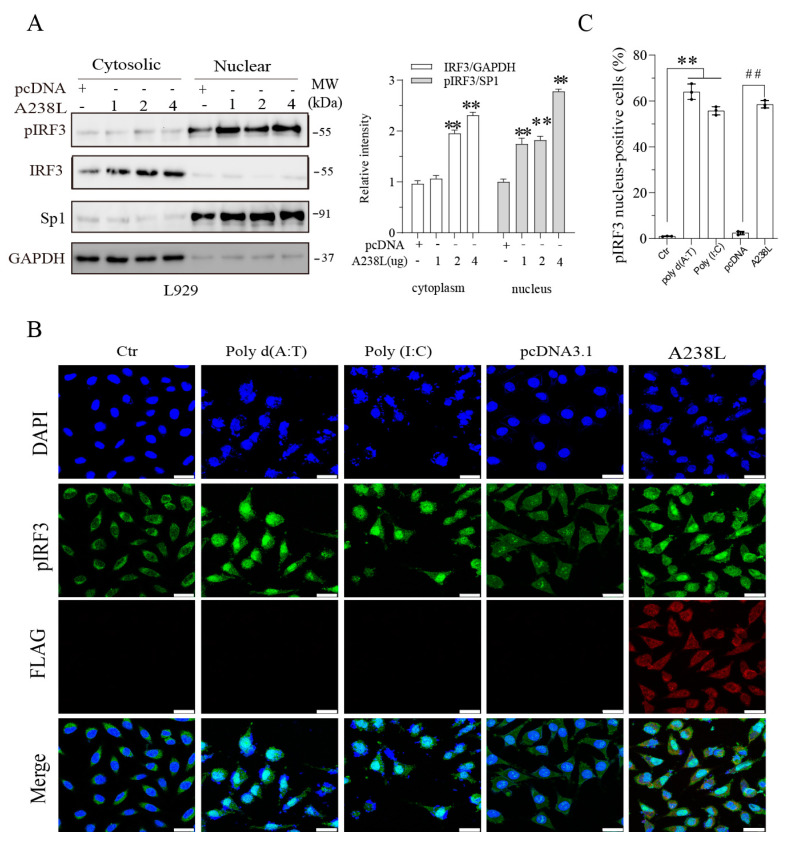
A238L promotes IRF3 nuclear translocation. (**A**) L929 cells were transfected with pcDNA (2 μg) or A238L (1, 2, or 4 μg) plasmids and then incubated for 48 h. Cytosolic and nuclear fractions were analyzed for TBK1 and IRF3 phosphorylation and then re-probed for their total proteins by Western blot. GAPDH and Sp1 were detected as a loading control of cytosolic and nuclear fractions, respectively. Relative IRF3 and phosphorylated IRF3 levels were semi-quantified using Image-J software. The results are presented as bar graphs. ** *p* < 0.01, compared to the pcDNA control. (**B**) L929 cells were transfected with pcDNA or A238L and incubated for 48 h or transfected with poly d (A:T), poly (I:C) and incubated for 12 h. The cells were fixed and analyzed for pIRF3 and A238L expression by immunofluorescence with an anti-phosphorylated IRF3 and anti-FLAG antibody. (**C**) The percentage of the cells with the phosphorylated IRF3 nuclear staining was calculated. The results are the means ± SD of three independent experiments. **, ## *p* < 0.01.

Phosphorylated IRF3 is translocated into the nucleus where it functions as a transcription factor to induce IFN gene expression [24]. A238L increased the levels of phosphorylated IRF3 protein in the nucleus. A238L increased the levels of unphosphorylated IRF3 in the cytoplasm in a dose-dependent manner (Figure 5A and Figures S3–S7). Of note, only a small fraction of IRF3, which was phosphorylated, was present in the nucleus. Immunofluorescence (IF) staining revealed that A238L, poly d (A:T), and poly (I:C) significantly increased the number of cells with nuclear pIRF3, compared to the mock- or pcDNA3.1-transfected control (Figure 5B,C).

## 4. Discussion

Widespread ASF epidemics over the past few years have instigated strong interest in understanding the function of uncharacterized or previously poorly characterized ASFV genes. For example, recent studies show that many ASFV genes such as EP364R, pI215L, and A137R can block the activation of the cGAS–STING pathway and inhibit IRF3 activity and IFN-I production [25,26,27,28]. ASFV can participate in evading the host immune response through multiple regulatory mechanisms. A variety of structural and nonstructural proteins of ASFV are involved in evading the host immune response. ASFV EP153R evades the host immune response by inhibiting the expression of major histocompatibility complex class I (MHC-I) [29]. CD2v promotes virus spread by adsorption to erythrocytes [30]. A238L, a relatively well-studied nonstructural protein, inhibits NF-κB activation and inflammatory cytokine production by inhibiting p300 activation [14]. Whether A238L affects the TBK1-IRF3 pathway and regulates innate immunity remains unknown. Our present study confirmed the inhibitory effect of A238L on NF-κB but unexpectedly found that A238L was able to activate the TBK1-IRF3 axis and induce IFN-β and ISG gene expression. Mechanistically, A238L activated the TBK1-IRF3 axis and increased TBK1 phosphorylation. Our study reveals a previously unanticipated role of A238L in enhancing antiviral immunity.

Upon sensing cytosolic dsDNA, cGAS binds to STING and then recruits TBK1 onto the endoplasmic reticulum [31]. Similarly, upon sensing viral RNA, RIG-I binds to MAVS in mitochondria to activate TBK1 and IKKε [32,33]. Activated TBK1 recruits and phosphorylates IRF3. Phosphorylated IRF3 becomes dimerized and is translocated to the nucleus where it functions to transcribe the IFN-I genes [34]. Several ASFV genes suppress cGAS–STING pathway activation. For example, DP96R inhibits cGAS–STING pathway activation, leading to the suppression of TBK1 and IRF3 phosphorylation and the downregulation of IFN production [35]. I329L inhibits dsRNA-mediated NF-κB and IRF3 activation and IFN production in 293T cells, probably by interfering with TRIF activity [36]. E120R interacts with IRF3, prevents its recruitment to TBK1, and inhibits IRF3 phosphorylation, leading to decreased interferon production [37]. Recently, Li et al. showed that MGF-505 inhibits the cGAS–STING pathway by increasing ULK1 expression and promoting autophagy-mediated cGAS degradation [38]. In contrast to these observations, we found that A238L increased TBK1 and IRF3 phosphorylation as well as IRF3 nuclear localization. A238L increased IRF3- and IFN-β-promoter-driven luciferase expression, IFN-β and ISG gene transcription, and antiviral activity. These findings suggest that A238L did not inhibit but rather enhanced the activation of the TBK1–IRF3 pathway. It should be noted that the activation of innate immunity by viral proteins is not unprecedented. Several enveloped viruses, such as respiratory syncytial virus, hepatitis C virus, measles virus, HIV, and coronavirus, encode proteins that bind and activate the TLR pathway [39].

NF-κB plays an essential role in transcribing a variety of genes involved in the inflammatory response [40,41,42]. Recent studies have shown that several ASFV genes inhibit NF-κB activation. For example, MGF360-12L interferes with NF-κB nuclear translocation by blocking its interaction with Importin α and inhibits type I IFN production [43]. ASFV ubiquitin-conjugating enzyme UBCv1 inhibits inflammatory signaling through NF-κB and AP-1 [44]. A238L shares homology with IκBα and binds to the p65 subunit of NF-κB to prevent its activation [45]. In addition, A238L disrupts p300 interaction with PKC-θ and represses p300-mediated NF-κB transactivation [17,46]. Our present study shows that A238L inhibited the expression of the pro-inflammatory cytokines, TNF-α, IL-6, and IL-1β. Thus, ASFV dampens the anti-inflammatory response by inactivating NF-κB via multiple genes through different mechanisms.

We are aware of a couple of weaknesses in our current study. First, whether A238L deletion in ASFV would downregulate the antiviral response was not investigated. However, this cannot be tested since the TBK-IRF3 pathway is not activated anyway by pathogenic ASFV strains [47]. Nevertheless, our study provides evidence that A238L inhibited NF-κB activation and downregulated the expression of several inflammatory cytokine genes. In contrast, A238L enhanced the activation of the TBK1-IRF3 pathway, leading to increased IFN-βand ISG gene expression and antiviral activity. Our study unveils an unanticipated function of the A238L gene in enhancing innate immunity.

## 5. Conclusions

A238L inhibits the activation of NF-κB by suppressing the HAT activity of p300. Whether A238L also affects the transcriptional activity of IRF3 remains unexplored. Here we first confirmed the ability of A238L to suppress NF-κB-activity in L929 cells. In contrast, A238L increased TBK1 and IRF3 phosphorylation and enhanced innate antiviral immunity in the absence or presence of poly d (A:T) or poly (I:C) stimulation, or herpes simplex virus type 1 (HSV-1) or Sendai virus (SeV) infection. This study reveals an unrecognized role for A238L in promoting antiviral immune responses by activating the TBK1-IRF3 pathway.

## Figures and Tables

**Table 1:** The primers for RT-PCR.

Primer	Sequence
TNF-α-F	CCCTCACACTCAGATCATCTTCT
TNF-α-R	GCTACGACGTGGGCTACAG
IL-6-F	TGAGATCTACTCGGCAAACCTAGTG
IL-6-R	CTTCGTAGAGAACAACATAAGTCAGATACC
IL-1β-F	TGGACCTTCCAGGATGAGGACA
IL-1β-R	TTCATCTCGGAGCCTGTAGTG
IFN-β-F	CAGCTCCAAGAAAGGACGAAC
IFN-β-R	GGCAGTGTAACTCTTCTGCAT
ISG56-F	TAGCCAACATGTCCTCACAGAC
ISG56-R	TCTTCTACCACTGGTTTCATGC
Mx1-F	GACCATAGGGGTCTTGACCAA
Mx1-R	AGACTTGCTCTTTCTGAAAAGCC
Actin-F	CATCCGTAAAGACCTCTATGCCAAC
Actin-R	ATGGAGCCACCGATCCACA

## Data Availability

The datasets generated during and/or analyzed during the current study are available from the corresponding author upon reasonable request.

## Supplementary Materials

The following supporting information can be downloaded at: https://www.mdpi.com/article/10.3390/vetsci11060252/s1.
