# Supplementary material for: Nonstructural Protein A238L of the African Swine Fever Virus (ASFV) Enhances Antiviral Immune Responses by Activating the TBK1-IRF3 Pathway
Source: Vet Sci. 2024 Jun 4;11(6):252. doi: 10.3390/vetsci11060252 (PMC11209439; doi:10.3390/vetsci11060252)
Supplement: Supplementary file 1 [file vetsci-11-00252-s001.zip › vetsci-2989088-Supplementary Materials.pdf]

Supplementary Materials

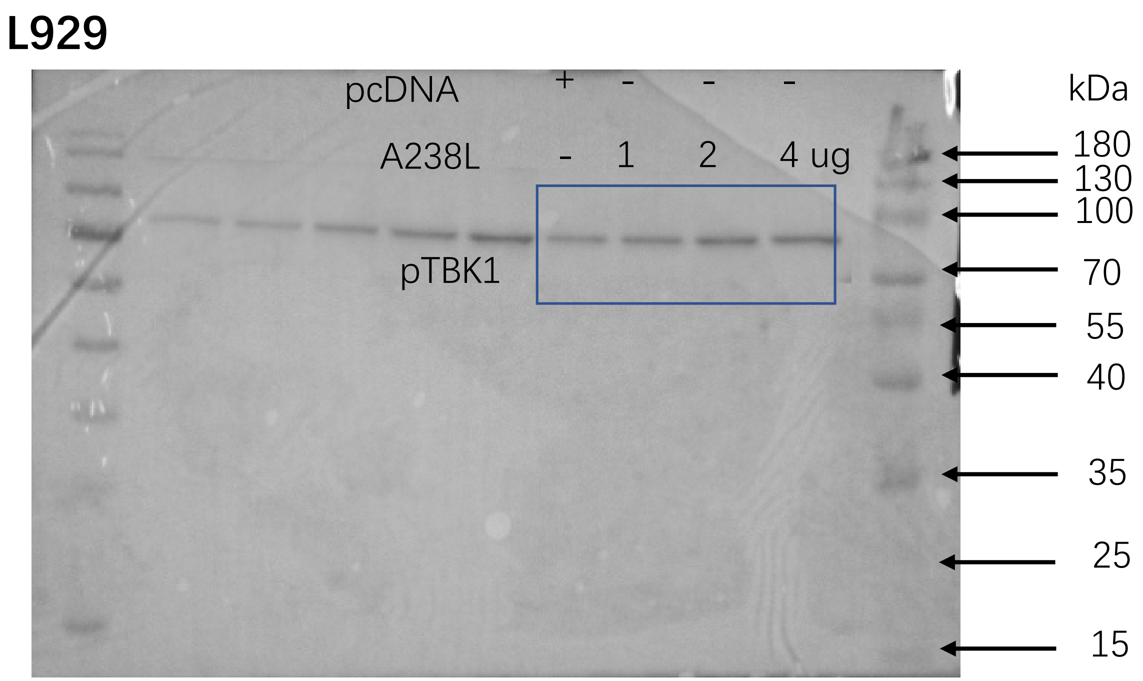

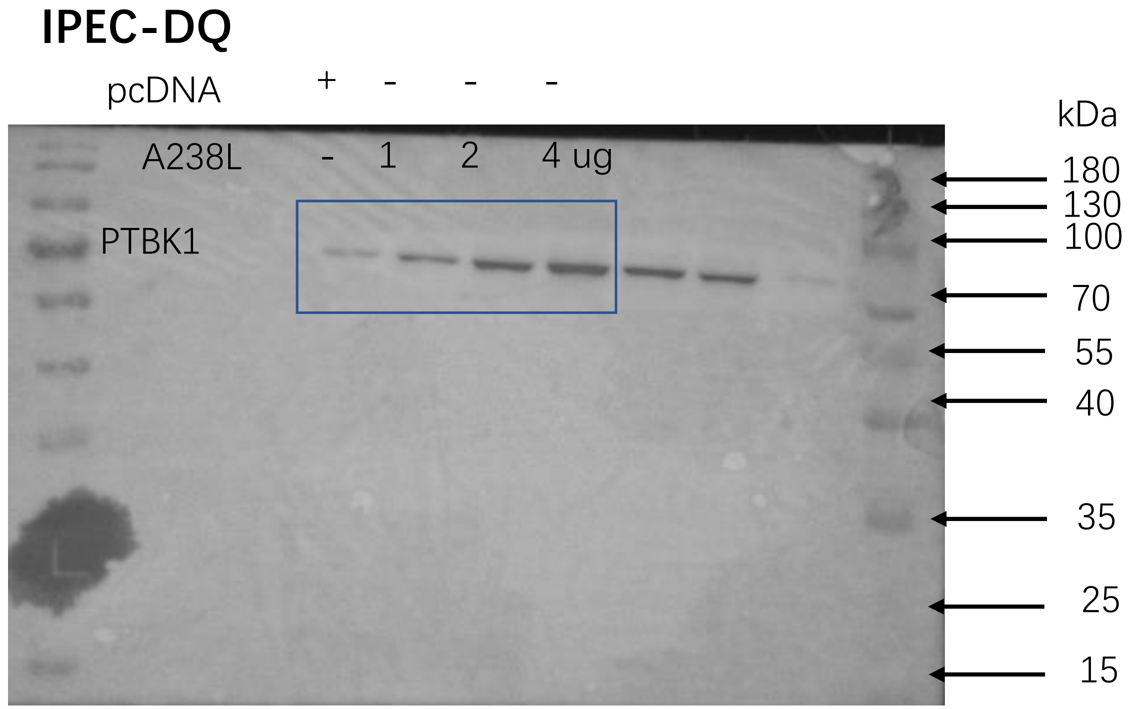

Figure S1. Western blot membrane of pTBK1 (~84 kDa) protein detected with anti-phosphorylated TBK1 (Ser172) (Cat# 4947; 1:1000; Cell Signaling Technology) antibody. Gel-separated proteins were transferred to nitrocellulose membranes (0.2 μm pore size; Bio-Rad, Hercules, USA) by semidry electroblotting (1.5 mA per cm2, 20 min). Membranes, incubated with a horseradish peroxidase-conjugated secondary antibody (AS09 602; 1:5000–1:10000; Agrisera), were developed with Pierce™ DAB Substrate Kit (Thermo Fisher Scientific). #Weight marker (molecular weight in kDa): Vazyme Prestained Protein Ladder, 10 to 180 kDa; catalogue number: MP102-01. Blot images, prior to the densitometry readings, were converted to grayscale with ImageJ (ImageJ v.1.49, National Institutes of Health, Maryland, USA) .

3D4/21

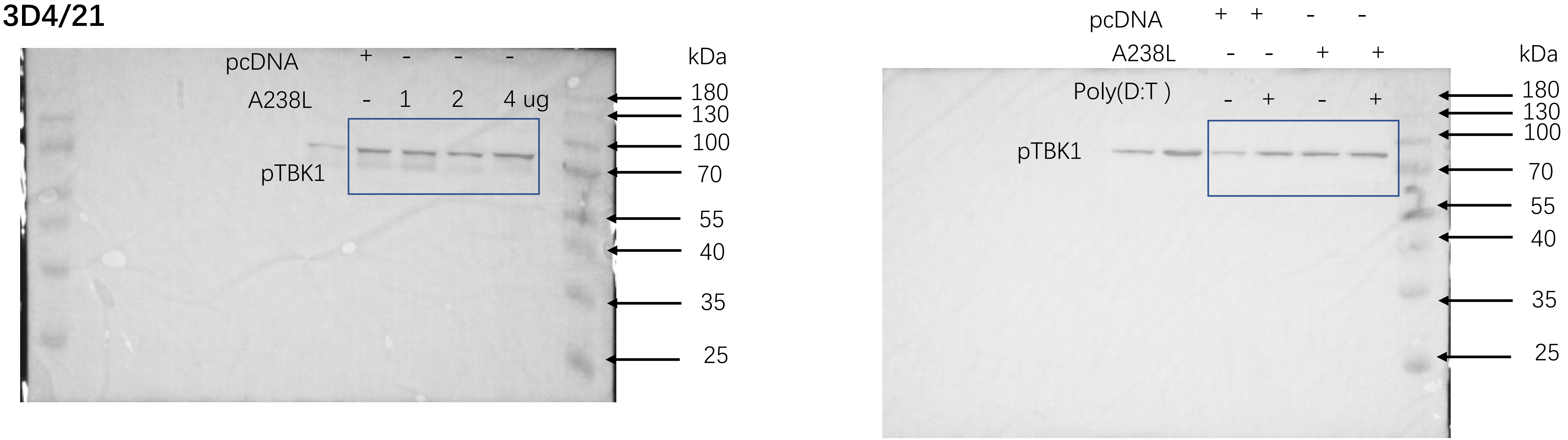

Figure S1. Western blot membrane of pTBK1 (~84 kDa) protein detected with anti-phosphorylated TBK1 (Ser172) (Cat# 4947; 1:1000; Cell Signaling Technology) antibody. Gel-separated proteins were transferred to nitrocellulose membranes (0.2 μm pore size; Bio-Rad, Hercules, USA) by semidry electroblotting (1.5 mA per cm2, 20 min). Membranes, incubated with a horseradish peroxidase-conjugated secondary antibody (AS09 602; 1:5000–1:10000; Agrisera), were developed with Pierce™ DAB Substrate Kit (Thermo Fisher Scientific). #Weight marker (molecular weight in kDa): Vazyme Prestained Protein Ladder, 10 to 180 kDa; catalogue number: MP102-01. Blot images, prior to the densitometry readings, were converted to grayscale with ImageJ (ImageJ v.1.49, National Institutes of Health, Maryland, USA) .

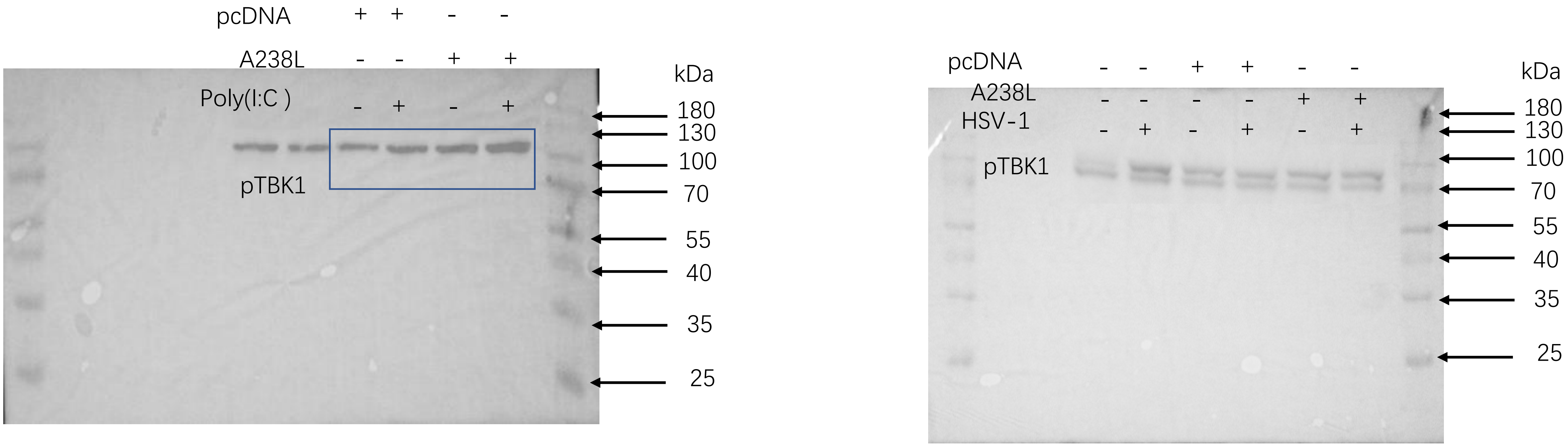

Figure S1. Western blot membrane of pTBK1 (~84 kDa) protein detected with anti-phosphorylated TBK1 (Ser172) (Cat# 4947; 1:1000; Cell Signaling Technology) antibody. Gel-separated proteins were transferred to nitrocellulose membranes (0.2 µm pore size; Bio-Rad, Hercules, USA) by semidry electroblotting (1.5 mA per cm2, 20 min). Membranes, incubated with a horseradish peroxidase-conjugated secondary antibody (AS09 602; 1:5000–1:10000; Agrisera), were developed with Pierce™ DAB Substrate Kit (Thermo Fisher Scientific). #Weight marker (molecular weight in kDa): Vazyme Prestained Protein Ladder, 10 to 180 kDa; catalogue number: MP102-01. Blot images, prior to the densitometry readings, were converted to grayscale with ImageJ (ImageJ v.1.49, National Institutes of Health, Maryland, USA) .

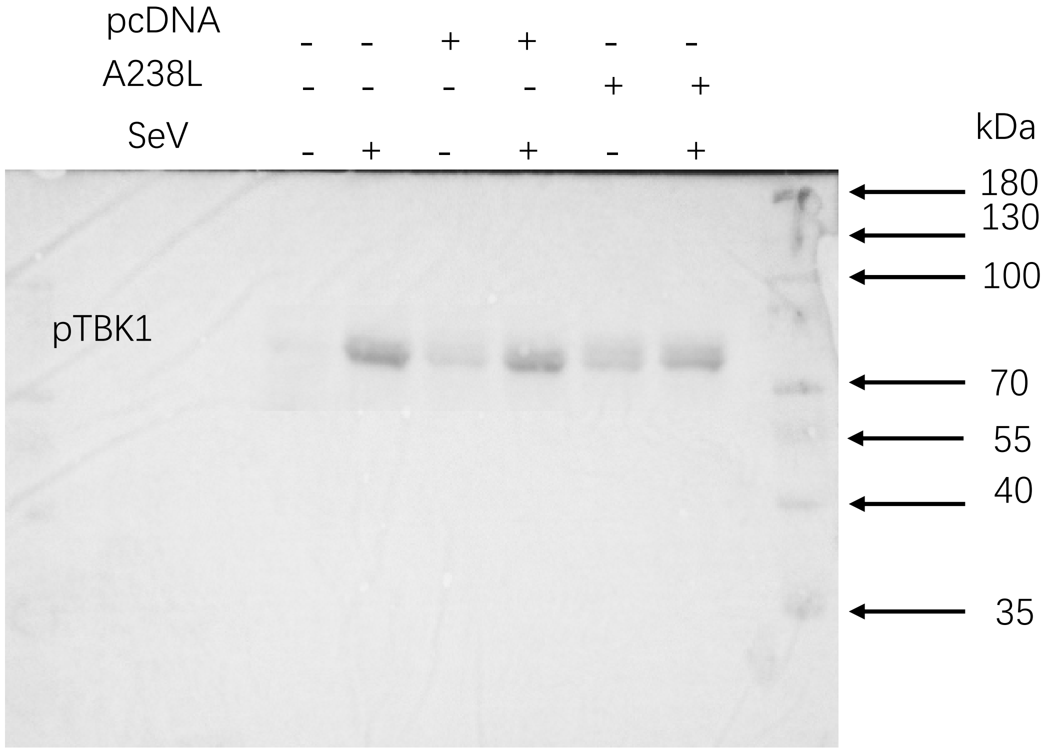

Figure S1. Western blot membrane of pTBK1 (~84 kDa) protein detected with anti-phosphorylated TBK1 (Ser172) (Cat# 4947; 1:1000; Cell Signaling Technology) antibody. Gel-separated proteins were transferred to nitrocellulose membranes (0.2 µm pore size; Bio-Rad, Hercules, USA) by semidry electroblotting (1.5 mA per cm2, 20 min). Membranes, incubated with a horseradish peroxidase-conjugated secondary antibody (AS09 602; 1:5000–1:10000; Agrisera), were developed with Pierce™ DAB Substrate Kit (Thermo Fisher Scientific). #Weight marker (molecular weight in kDa): Vazyme Prestained Protein Ladder, 10 to 180 kDa; catalogue number: MP102-01. Blot images, prior to the densitometry readings, were converted to grayscale with ImageJ (ImageJ v.1.49, National Institutes of Health, Maryland, USA) .

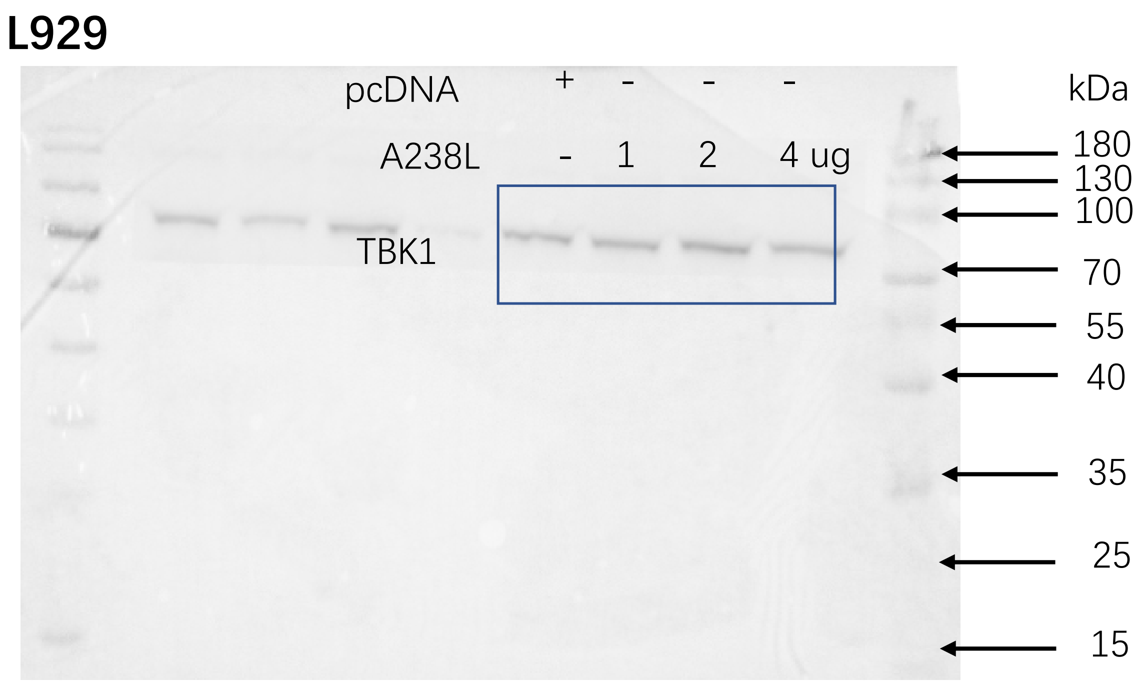

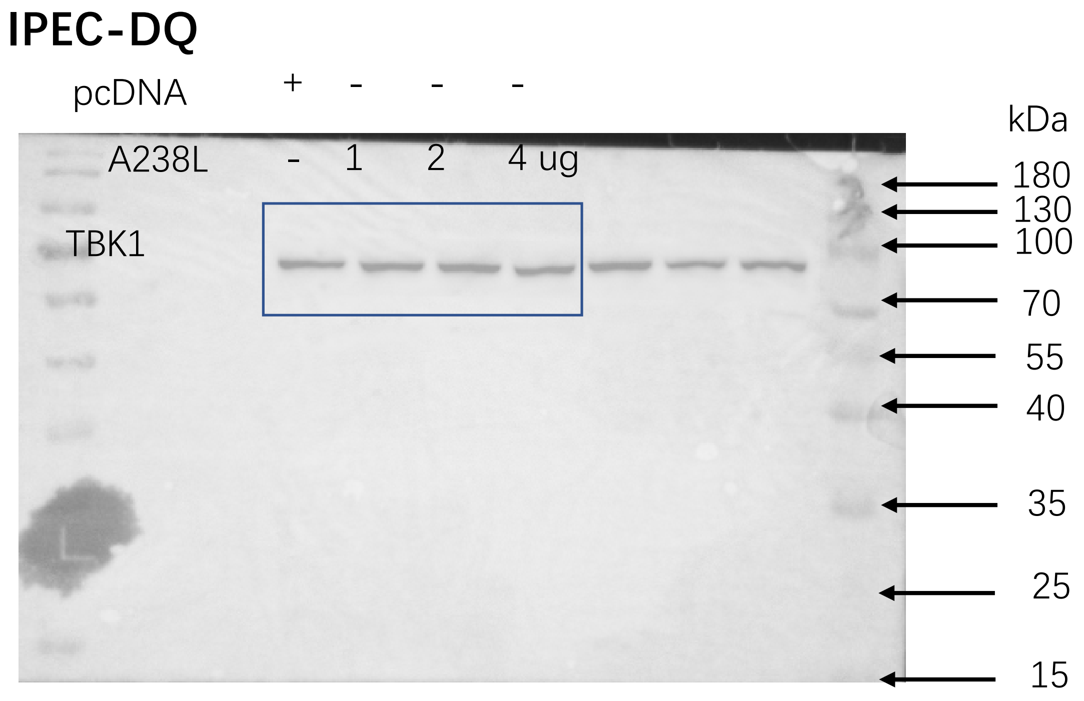

Figure S2. Western blot membrane of TBK1 (~84 kDa) protein detected with TBK1 (Cat# 3504; 1:1000; Cell Signaling Technology) antibody. Gel-separated proteins were transferred to nitrocellulose membranes (0.2 µm pore size; Bio-Rad, Hercules, USA) by semidry electroblotting (1.5 mA per cm2, 20 min). Membranes, incubated with a horseradish peroxidase-conjugated secondary antibody (AS09 602; 1:5000–1:10000; Agrisera), were developed with Pierce™ DAB Substrate Kit (Thermo Fisher Scientific). #Weight marker (molecular weight in kDa): Vazyme Prestained Protein Ladder, 10 to 180 kDa; catalogue number: MP102-01. Blot images, prior to the densitometry readings, were converted to grayscale with ImageJ (ImageJ v.1.49, National Institutes of Health, Maryland, USA) .

3D4/21

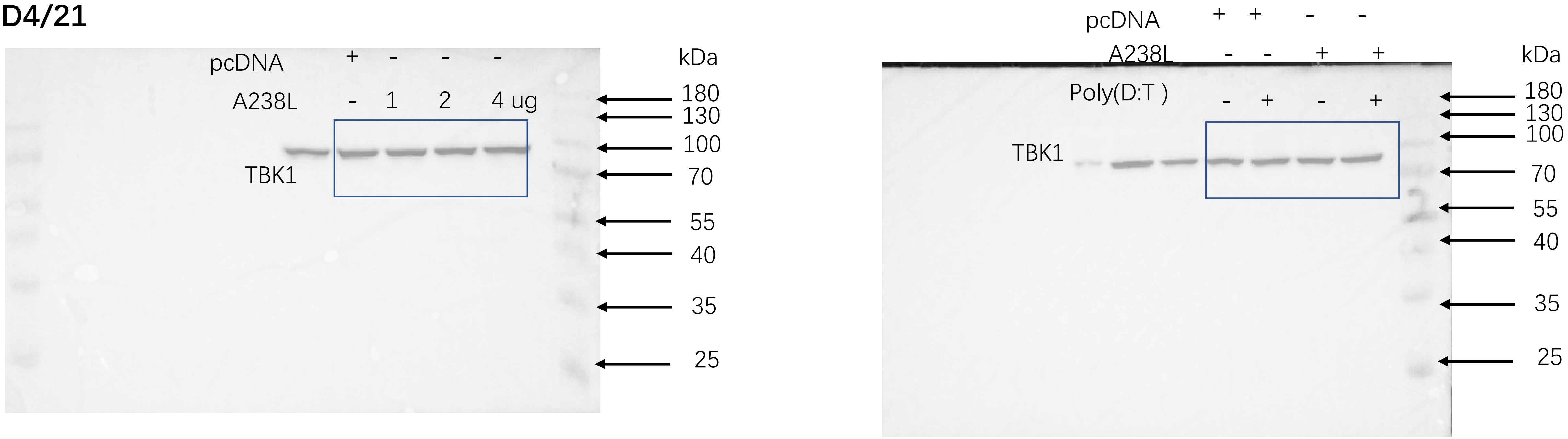

Figure S2. Western blot membrane of TBK1 (~84 kDa) protein detected with TBK1 (Cat# 3504; 1:1000; Cell Signaling Technology) antibody. Gel-separated proteins were transferred to nitrocellulose membranes (0.2 μm pore size; Bio-Rad, Hercules, USA) by semidry electroblotting (1.5 mA per cm2, 20 min). Membranes, incubated with a horseradish peroxidase-conjugated secondary antibody (AS09 602; 1:5000–1:10000; Agrisera), were developed with Pierce™ DAB Substrate Kit (Thermo Fisher Scientific). #Weight marker (molecular weight in kDa): Vazyme Prestained Protein Ladder, 10 to 180 kDa; catalogue number: MP102-01. Blot images, prior to the densitometry readings, were converted to grayscale with ImageJ (ImageJ v.1.49, National Institutes of Health, Maryland, USA) .

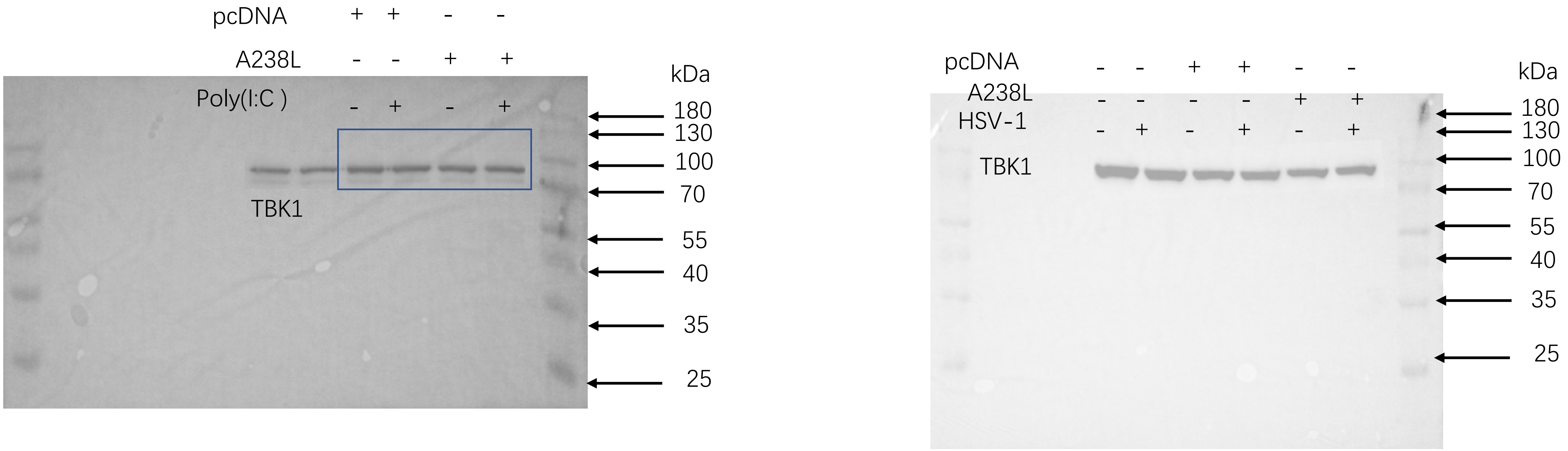

Figure S2. Western blot membrane of TBK1 (~84 kDa) protein detected with TBK1 (Cat# 3504; 1:1000; Cell Signaling Technology) antibody. Gel-separated proteins were transferred to nitrocellulose membranes (0.2 μm pore size; Bio-Rad, Hercules, USA) by semidry electroblotting (1.5 mA per cm2, 20 min). Membranes, incubated with a horseradish peroxidase-conjugated secondary antibody (AS09 602; 1:5000–1:10000; Agrisera), were developed with Pierce™ DAB Substrate Kit (Thermo Fisher Scientific). #Weight marker (molecular weight in kDa): Vazyme Prestained Protein Ladder, 10 to 180 kDa; catalogue number: MP102-01. Blot images, prior to the densitometry readings, were converted to grayscale with ImageJ (ImageJ v.1.49, National Institutes of Health, Maryland, USA) .

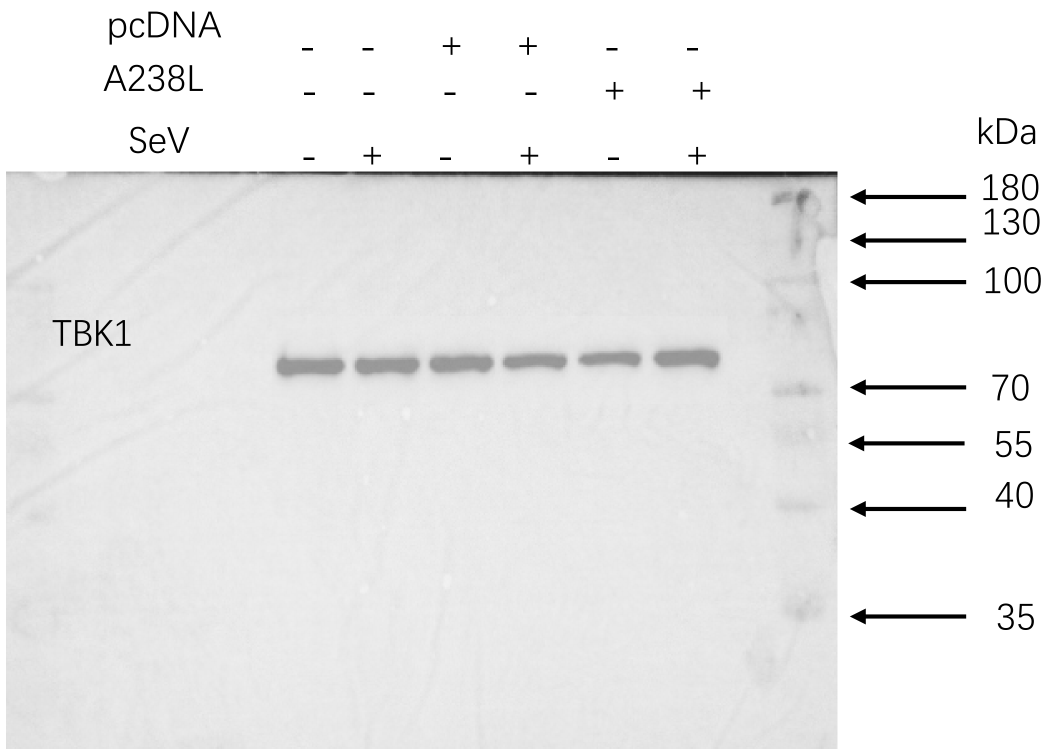

Figure S2. Western blot membrane of TBK1 (~84 kDa) protein detected with TBK1 (Cat# 3504; 1:1000; Cell Signaling Technology) antibody. Gel-separated proteins were transferred to nitrocellulose membranes (0.2 μm pore size; Bio-Rad, Hercules, USA) by semidry electroblotting (1.5 mA per cm2, 20 min). Membranes, incubated with a horseradish peroxidase-conjugated secondary antibody (AS09 602; 1:5000–1:10000; Agrisera), were developed with Pierce™ DAB Substrate Kit (Thermo Fisher Scientific). #Weight marker (molecular weight in kDa): Vazyme Prestained Protein Ladder, 10 to 180 kDa; catalogue number: MP102-01. Blot images, prior to the densitometry readings, were converted to grayscale with ImageJ (ImageJ v.1.49, National Institutes of Health, Maryland, USA) .

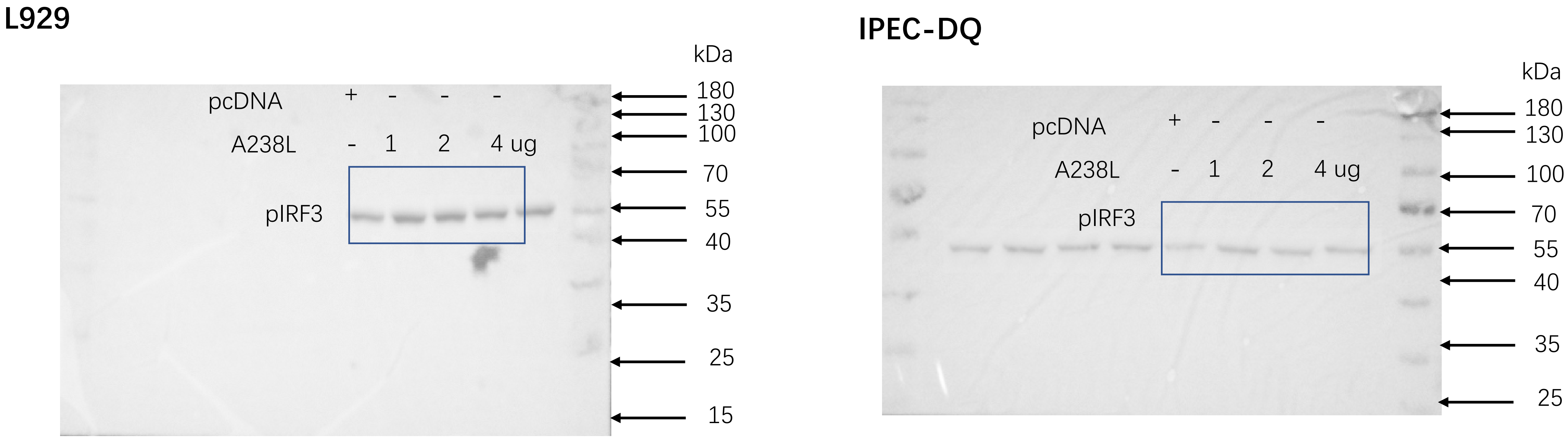

Figure S3. Western blot membrane of pIRF3 (~55 kDa) protein detected with phosphorylated IRF3 (Ser396) (Cat# 4947; 1:1000; Cell Signaling Technology) antibody. Gel-separated proteins were transferred to nitrocellulose membranes (0.2 μm pore size; Bio-Rad, Hercules, USA) by semidry electroblotting (1.5 mA per cm2, 20 min). Membranes, incubated with a horseradish peroxidase-conjugated secondary antibody (AS09 602; 1:5000–1:10000; Agrisera), were developed with Pierce™ DAB Substrate Kit (Thermo Fisher Scientific). #Weight marker (molecular weight in kDa): Vazyme Prestained Protein Ladder, 10 to 180 kDa; catalogue number: MP102-01. Blot images, prior to the densitometry readings, were converted to grayscale with ImageJ (ImageJ v.1.49, National Institutes of Health, Maryland, USA) .

3D4/21

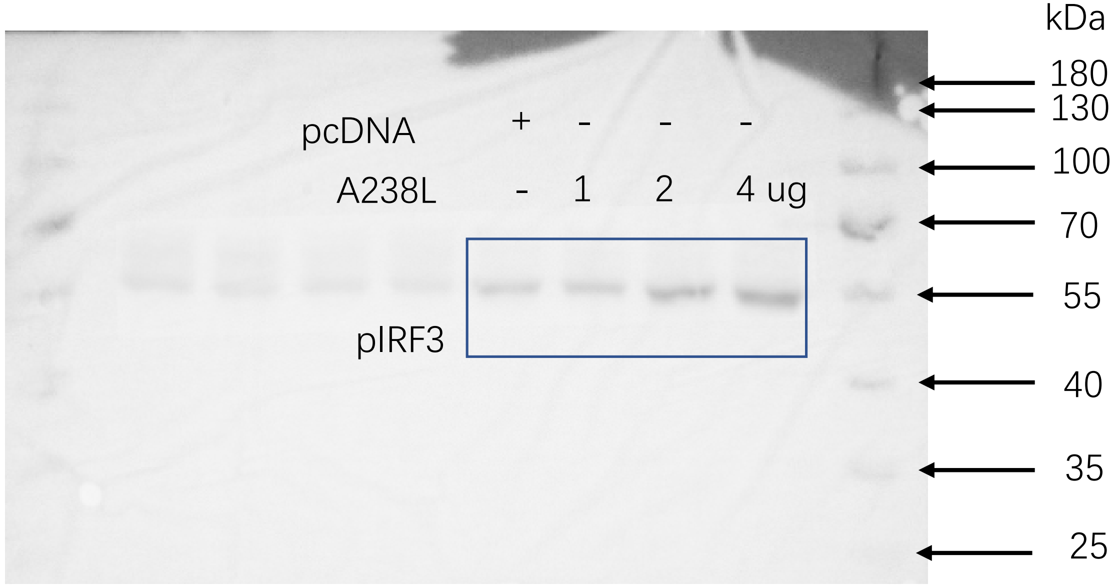

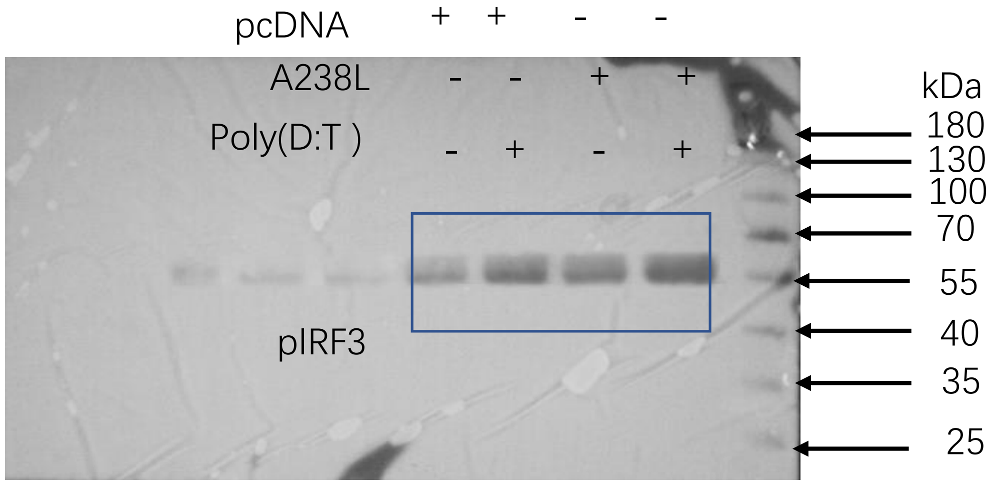

Figure S3. Western blot membrane of pIRF3 (~55 kDa) protein detected with phosphorylated IRF3 (Ser396) (Cat# 4947; 1:1000; Cell Signaling Technology) antibody. Gel-separated proteins were transferred to nitrocellulose membranes (0.2 µm pore size; Bio-Rad, Hercules, USA) by semidry electroblotting (1.5 mA per cm2, 20 min). Membranes, incubated with a horseradish peroxidase-conjugated secondary antibody (AS09 602; 1:5000–1:10000; Agrisera), were developed with Pierce™ DAB Substrate Kit (Thermo Fisher Scientific). #Weight marker (molecular weight in kDa): Vazyme Prestained Protein Ladder, 10 to 180 kDa; catalogue number: MP102-01. Blot images, prior to the densitometry readings, were converted to grayscale with ImageJ (ImageJ v.1.49, National Institutes of Health, Maryland, USA) .

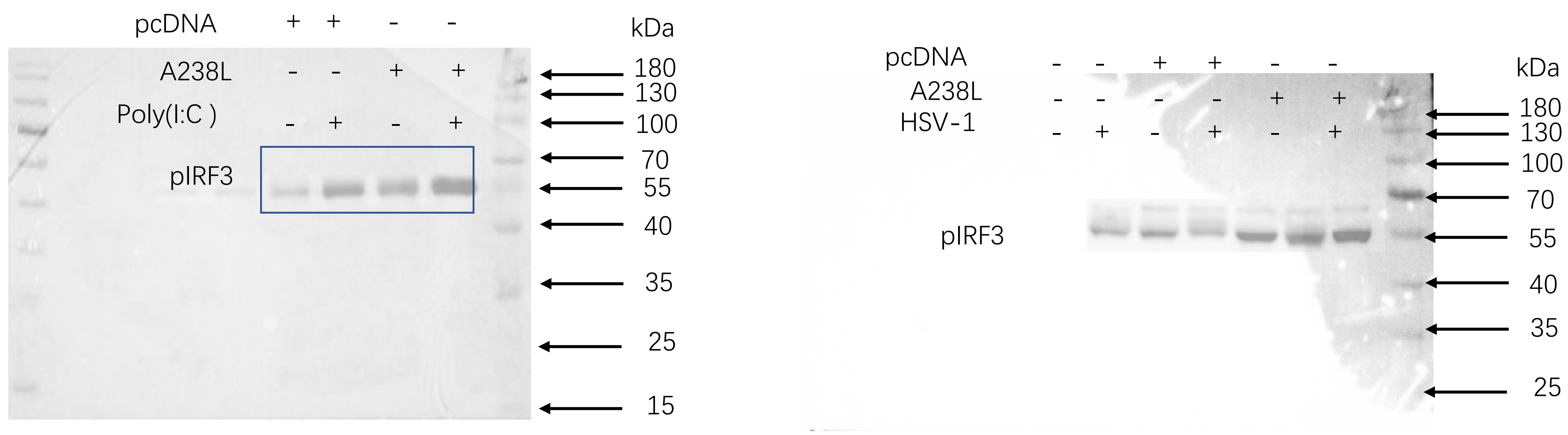

Figure S3. Western blot membrane of pIRF3 (~55 kDa) protein detected with phosphorylated IRF3 (Ser396) (Cat# 4947; 1:1000; Cell Signaling Technology) antibody. Gel-separated proteins were transferred to nitrocellulose membranes (0.2 µm pore size; Bio-Rad, Hercules, USA) by semidry electroblotting (1.5 mA per cm2, 20 min). Membranes, incubated with a horseradish peroxidase-conjugated secondary antibody (AS09 602; 1:5000–1:10000; Agrisera), were developed with Pierce™ DAB Substrate Kit (Thermo Fisher Scientific). #Weight marker (molecular weight in kDa): Vazyme Prestained Protein Ladder, 10 to 180 kDa; catalogue number: MP102-01. Blot images, prior to the densitometry readings, were converted to grayscale with ImageJ (ImageJ v.1.49, National Institutes of Health, Maryland, USA) .

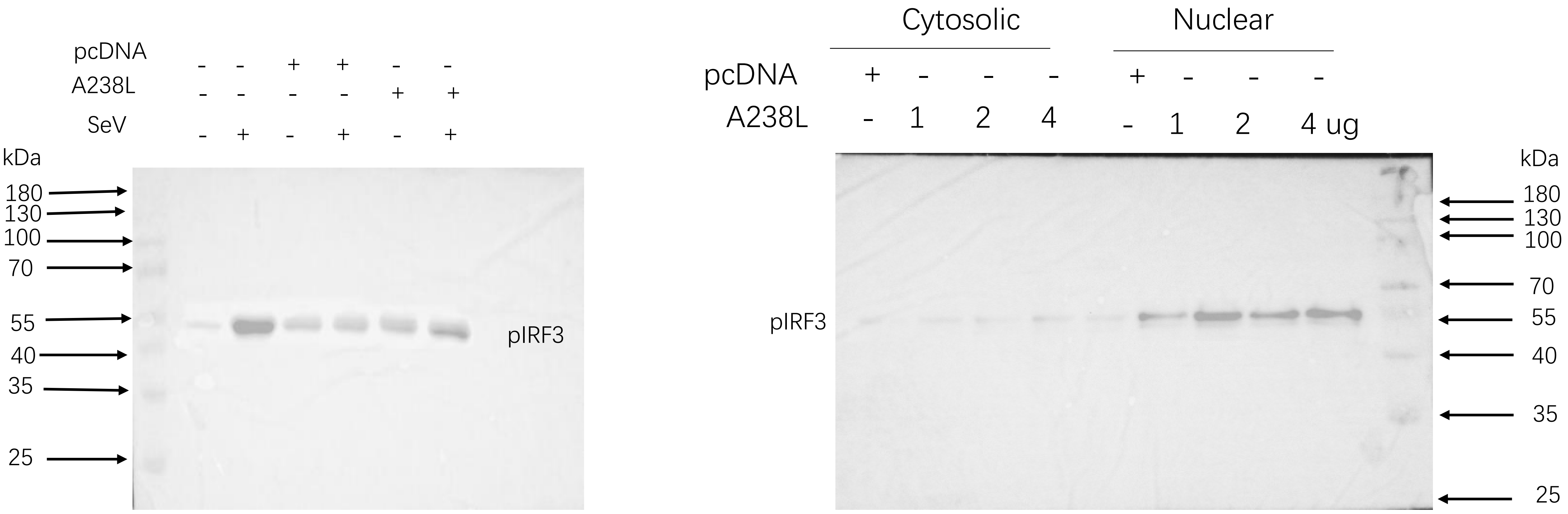

Figure S3. Western blot membrane of pIRF3 (~55 kDa) protein detected with phosphorylated IRF3 (Ser396) (Cat# 4947; 1:1000; Cell Signaling Technology) antibody. Gel-separated proteins were transferred to nitrocellulose membranes (0.2 µm pore size; Bio-Rad, Hercules, USA) by semidry electroblotting (1.5 mA per cm2, 20 min). Membranes, incubated with a horseradish peroxidase-conjugated secondary antibody (AS09 602; 1:5000–1:10000; Agrisera), were developed with Pierce™ DAB Substrate Kit (Thermo Fisher Scientific). #Weight marker (molecular weight in kDa): Vazyme Prestained Protein Ladder, 10 to 180 kDa; catalogue number: MP102-01. Blot images, prior to the densitometry readings, were converted to grayscale with ImageJ (ImageJ v.1.49, National Institutes of Health, Maryland, USA) .

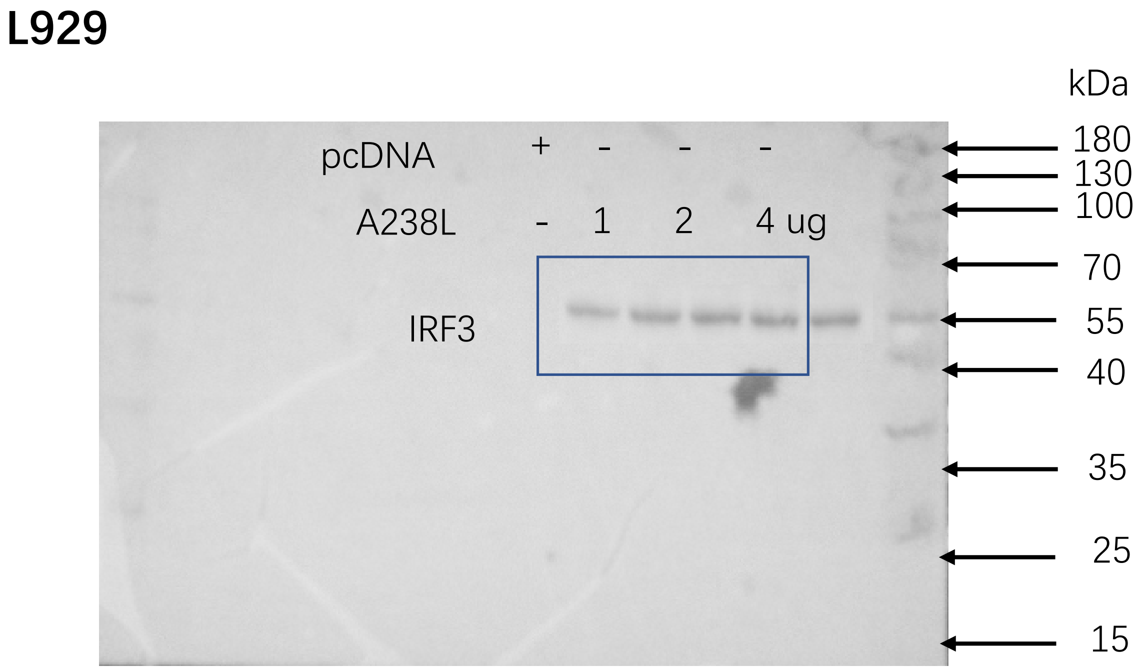

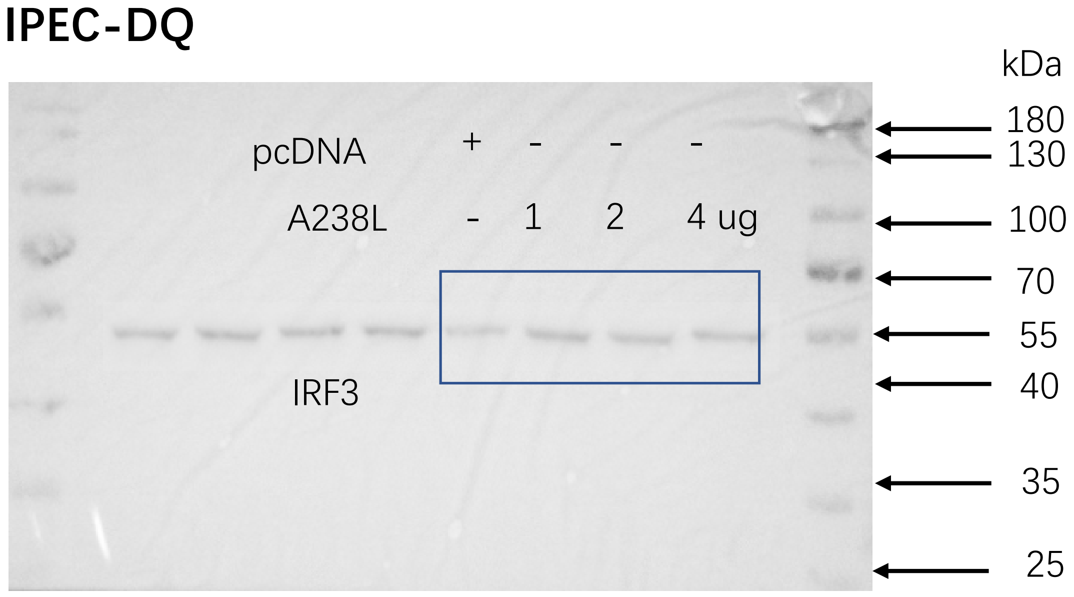

Figure S4. Western blot membrane of IRF3 (~55 kDa) protein detected with IRF3 (Cat# 4302; 1:1000; Cell Signaling Technology) antibody. Gel-separated proteins were transferred to nitrocellulose membranes (0.2 µm pore size; Bio-Rad, Hercules, USA) by semidry electroblotting (1.5 mA per cm2, 20 min). Membranes, incubated with a horseradish peroxidase-conjugated secondary antibody (AS09 602; 1:5000–1:10000; Agrisera), were developed with Pierce™ DAB Substrate Kit (Thermo Fisher Scientific). #Weight marker (molecular weight in kDa): Vazyme Prestained Protein Ladder, 10 to 180 kDa; catalogue number: MP102-01. Blot images, prior to the densitometry readings, were converted to grayscale with ImageJ (ImageJ v.1.49, National Institutes of Health, Maryland, USA) .

3D4/21

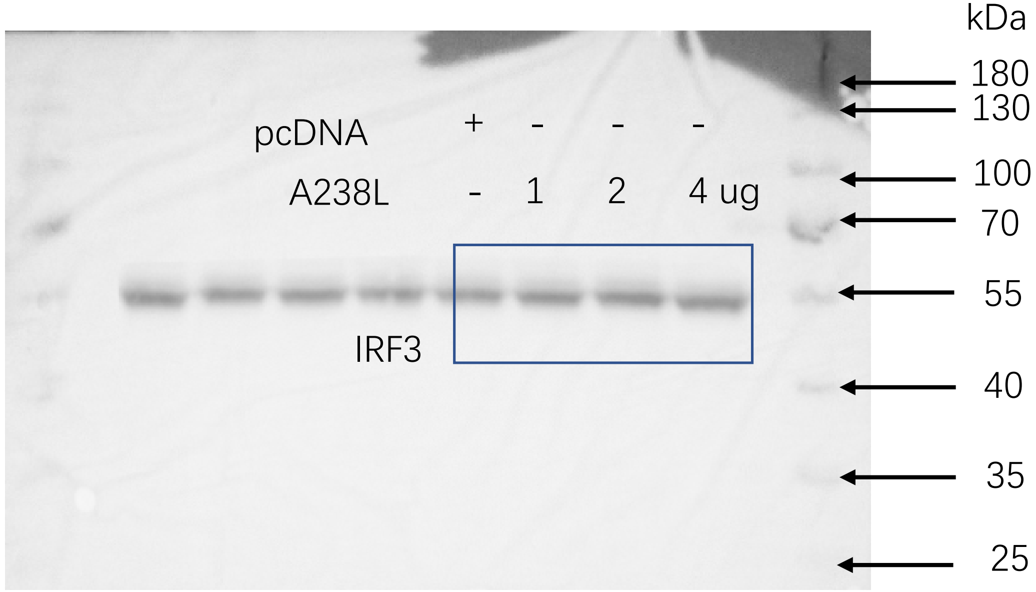

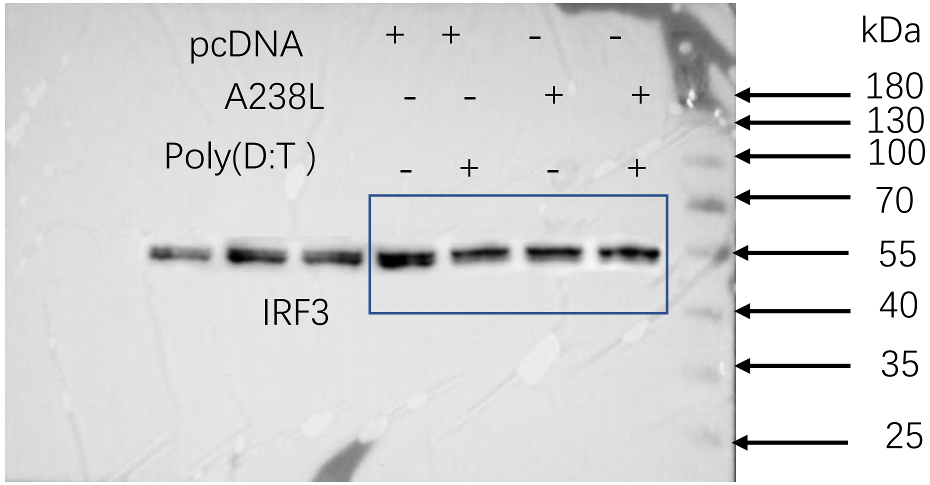

Figure S4. Western blot membrane of IRF3 (~55 kDa) protein detected with IRF3 (Cat# 4302; 1:1000; Cell Signaling Technology) antibody. Gel-separated proteins were transferred to nitrocellulose membranes (0.2 µm pore size; Bio-Rad, Hercules, USA) by semidry electroblotting (1.5 mA per cm2, 20 min). Membranes, incubated with a horseradish peroxidase-conjugated secondary antibody (AS09 602; 1:5000–1:10000; Agrisera), were developed with Pierce™ DAB Substrate Kit (Thermo Fisher Scientific). #Weight marker (molecular weight in kDa): Vazyme Prestained Protein Ladder, 10 to 180 kDa; catalogue number: MP102-01. Blot images, prior to the densitometry readings, were converted to grayscale with ImageJ (ImageJ v.1.49, National Institutes of Health, Maryland, USA) .

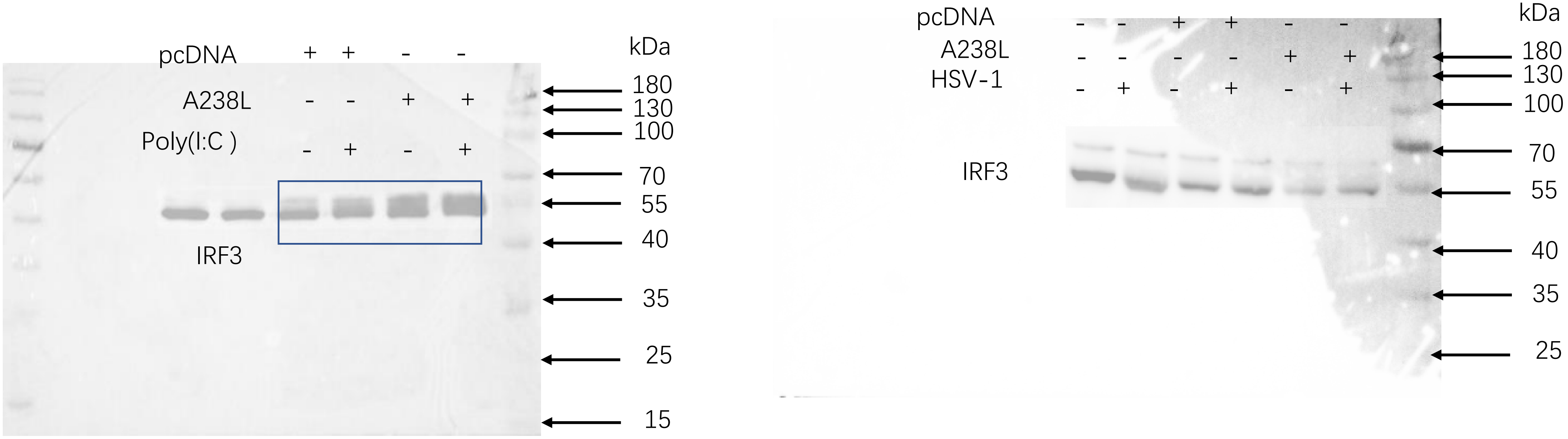

Figure S4. Western blot membrane of IRF3 (~55 kDa) protein detected with IRF3 (Cat# 4302; 1:1000; Cell Signaling Technology) antibody. Gel-separated proteins were transferred to nitrocellulose membranes (0.2 µm pore size; Bio-Rad, Hercules, USA) by semidry electroblotting (1.5 mA per cm2, 20 min). Membranes, incubated with a horseradish peroxidase-conjugated secondary antibody (AS09 602; 1:5000–1:10000; Agrisera), were developed with Pierce™ DAB Substrate Kit (Thermo Fisher Scientific). #Weight marker (molecular weight in kDa): Vazyme Prestained Protein Ladder, 10 to 180 kDa; catalogue number: MP102-01. Blot images, prior to the densitometry readings, were converted to grayscale with ImageJ (ImageJ v.1.49, National Institutes of Health, Maryland, USA) .

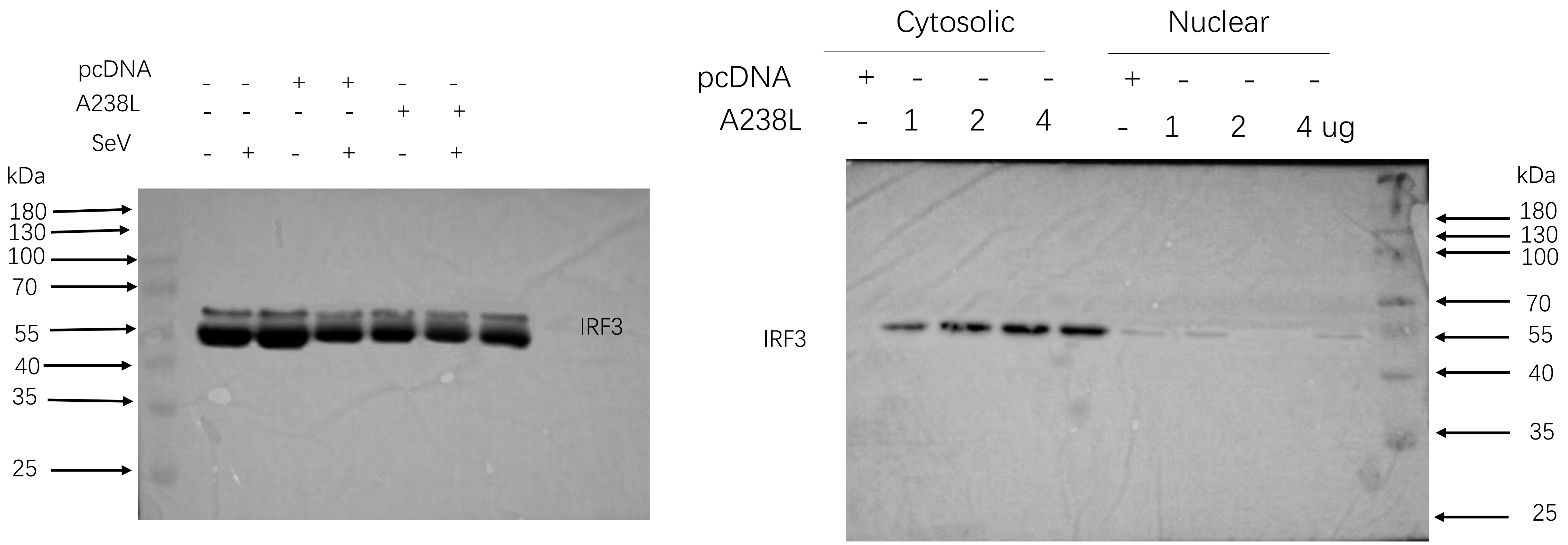

Figure S4. Western blot membrane of IRF3 (~55 kDa) protein detected with IRF3 (Cat# 4302; 1:1000; Cell Signaling Technology) antibody. Gel-separated proteins were transferred to nitrocellulose membranes (0.2 µm pore size; Bio-Rad, Hercules, USA) by semidry electroblotting (1.5 mA per cm2, 20 min). Membranes, incubated with a horseradish peroxidase-conjugated secondary antibody (AS09 602; 1:5000–1:10000; Agrisera), were developed with Pierce™ DAB Substrate Kit (Thermo Fisher Scientific). #Weight marker (molecular weight in kDa): Vazyme Prestained Protein Ladder, 10 to 180 kDa; catalogue number: MP102-01. Blot images, prior to the densitometry readings, were converted to grayscale with ImageJ (ImageJ v.1.49, National Institutes of Health, Maryland, USA) .

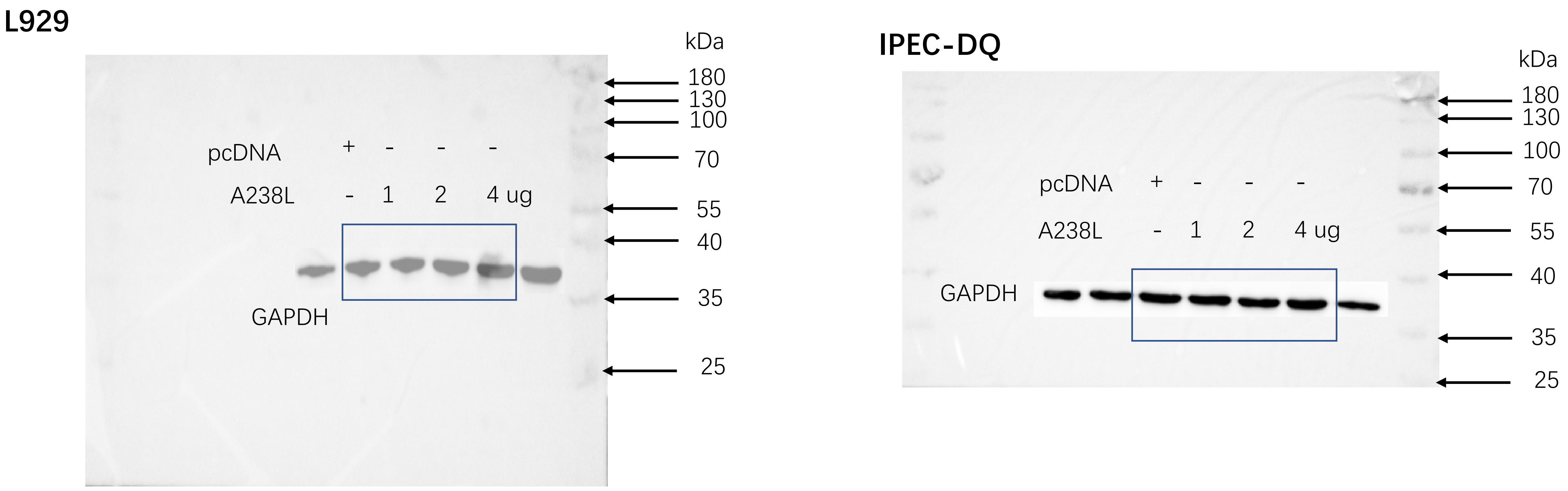

Figure S5. Western blot membrane of GAPDH (~37 kDa) protein detected with GAPDH (Cat# c-47724; 1:1000; Santa Cruz Biotechnology) antibody. Gel-separated proteins were transferred to nitrocellulose membranes (0.2 µm pore size; Bio-Rad, Hercules, USA) by semidry electroblotting (1.5 mA per cm2, 20 min). Membranes, incubated with a horseradish peroxidase-conjugated secondary antibody (AS09 602; 1:5000–1:10000; Agrisera), were developed with Pierce™ DAB Substrate Kit (Thermo Fisher Scientific). #Weight marker (molecular weight in kDa): Vazyme Prestained Protein Ladder, 10 to 180 kDa; catalogue number: MP102-01. Blot images, prior to the densitometry readings, were converted to grayscale with ImageJ (ImageJ v.1.49, National Institutes of Health, Maryland, USA) .

3D4/21

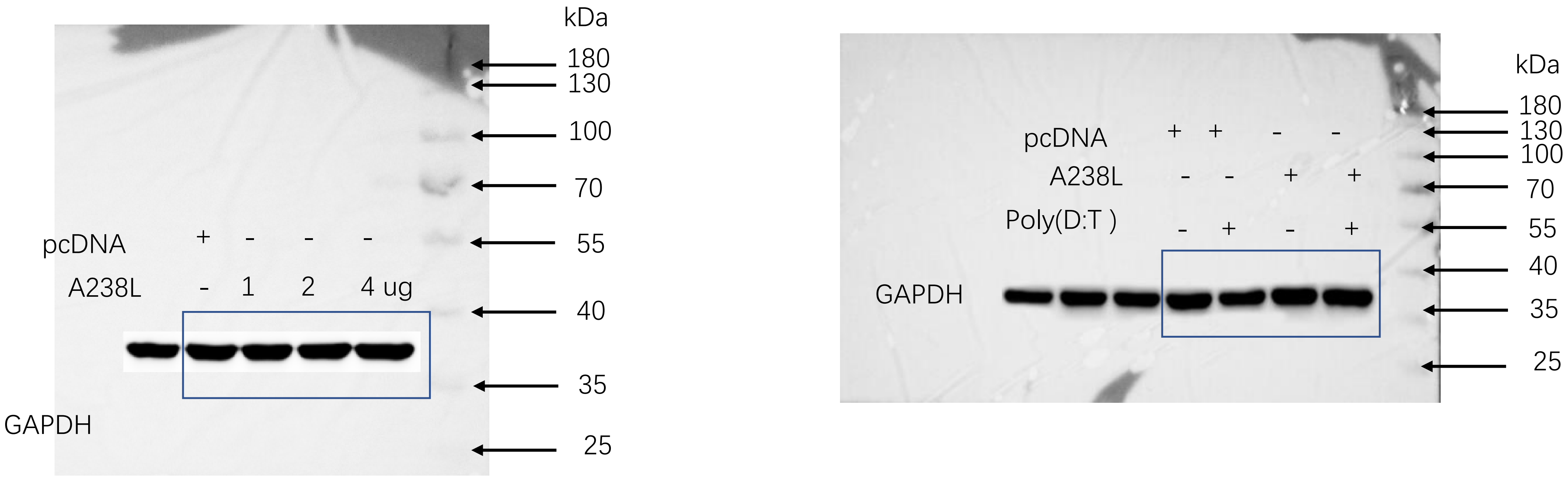

Figure S5. Western blot membrane of GAPDH (~37 kDa) protein detected with GAPDH (Cat# c-47724; 1:1000; Santa Cruz Biotechnology) antibody. Gel-separated proteins were transferred to nitrocellulose membranes (0.2 µm pore size; Bio-Rad, Hercules, USA) by semidry electroblotting (1.5 mA per cm2, 20 min). Membranes, incubated with a horseradish peroxidase-conjugated secondary antibody (AS09 602; 1:5000–1:10000; Agrisera), were developed with Pierce™ DAB Substrate Kit (Thermo Fisher Scientific). #Weight marker (molecular weight in kDa): Vazyme Prestained Protein Ladder, 10 to 180 kDa; catalogue number: MP102-01. Blot images, prior to the densitometry readings, were converted to grayscale with ImageJ (ImageJ v.1.49, National Institutes of Health, Maryland, USA) .

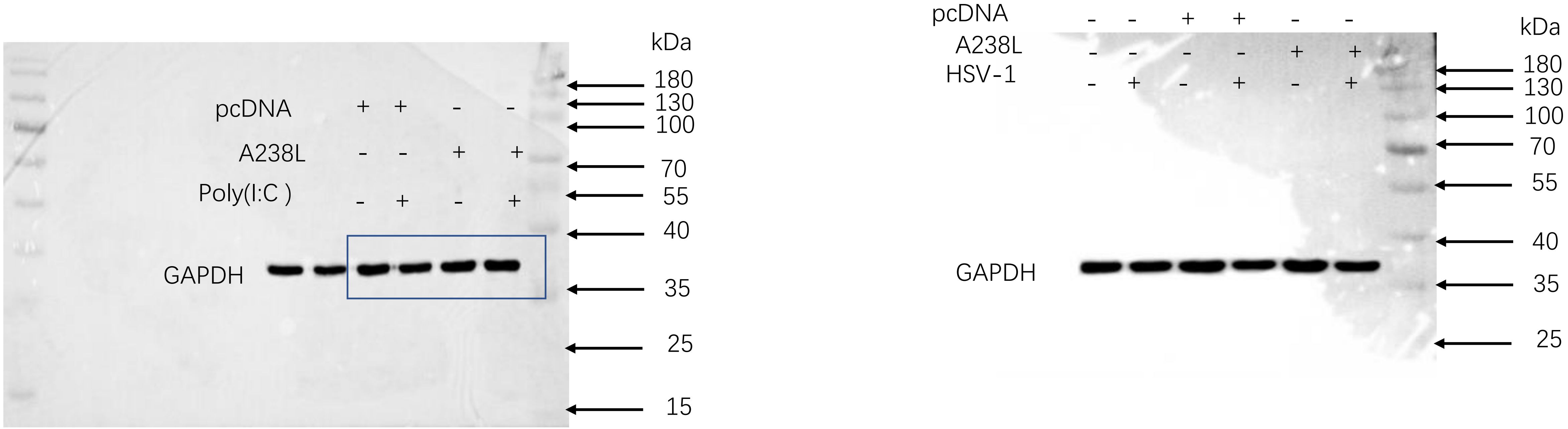

Figure S5. Western blot membrane of GAPDH (~37 kDa) protein detected with GAPDH (Cat# c-47724; 1:1000; Santa Cruz Biotechnology) antibody. Gel-separated proteins were transferred to nitrocellulose membranes (0.2 µm pore size; Bio-Rad, Hercules, USA) by semidry electroblotting (1.5 mA per cm2, 20 min). Membranes, incubated with a horseradish peroxidase-conjugated secondary antibody (AS09 602; 1:5000–1:10000; Agrisera), were developed with Pierce™ DAB Substrate Kit (Thermo Fisher Scientific). #Weight marker (molecular weight in kDa): Vazyme Prestained Protein Ladder, 10 to 180 kDa; catalogue number: MP102-01. Blot images, prior to the densitometry readings, were converted to grayscale with ImageJ (ImageJ v.1.49, National Institutes of Health, Maryland, USA) .

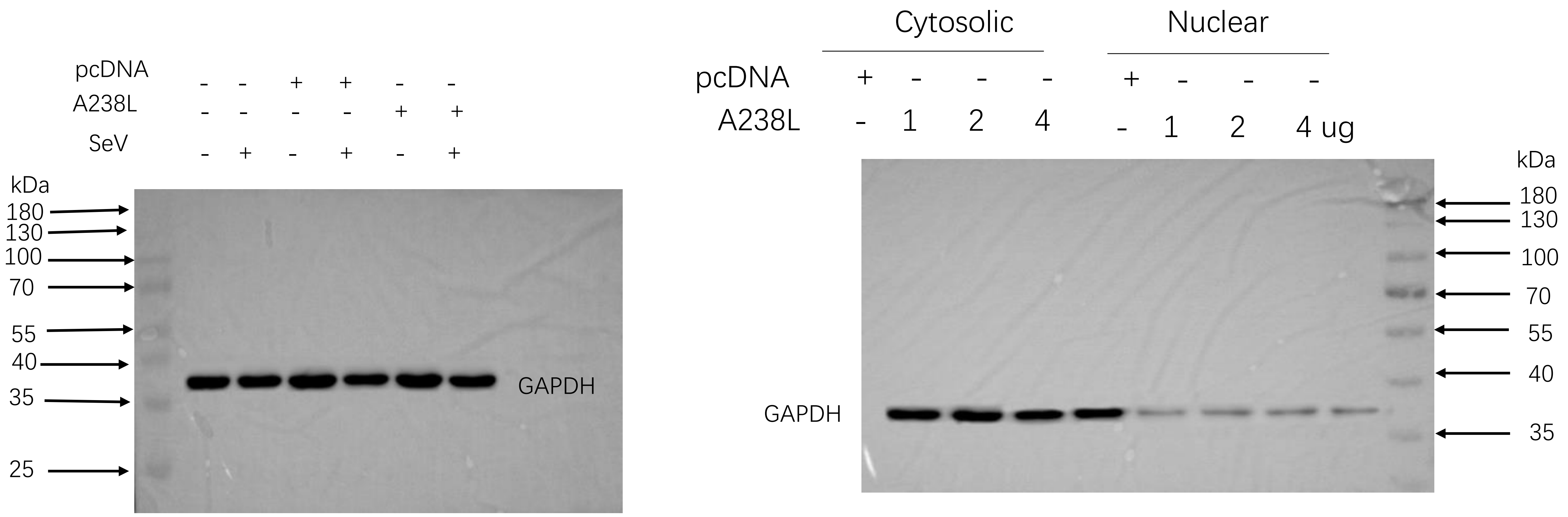

Figure S5. Western blot membrane of GAPDH (~37 kDa) protein detected with GAPDH (Cat# c-47724; 1:1000; Santa Cruz Biotechnology) antibody. Gel-separated proteins were transferred to nitrocellulose membranes (0.2 µm pore size; Bio-Rad, Hercules, USA) by semidry electroblotting (1.5 mA per cm2, 20 min). Membranes, incubated with a horseradish peroxidase-conjugated secondary antibody (AS09 602; 1:5000–1:10000; Agrisera), were developed with Pierce™ DAB Substrate Kit (Thermo Fisher Scientific). #Weight marker (molecular weight in kDa): Vazyme Prestained Protein Ladder, 10 to 180 kDa; catalogue number: MP102-01. Blot images, prior to the densitometry readings, were converted to grayscale with ImageJ (ImageJ v.1.49, National Institutes of Health, Maryland, USA) .

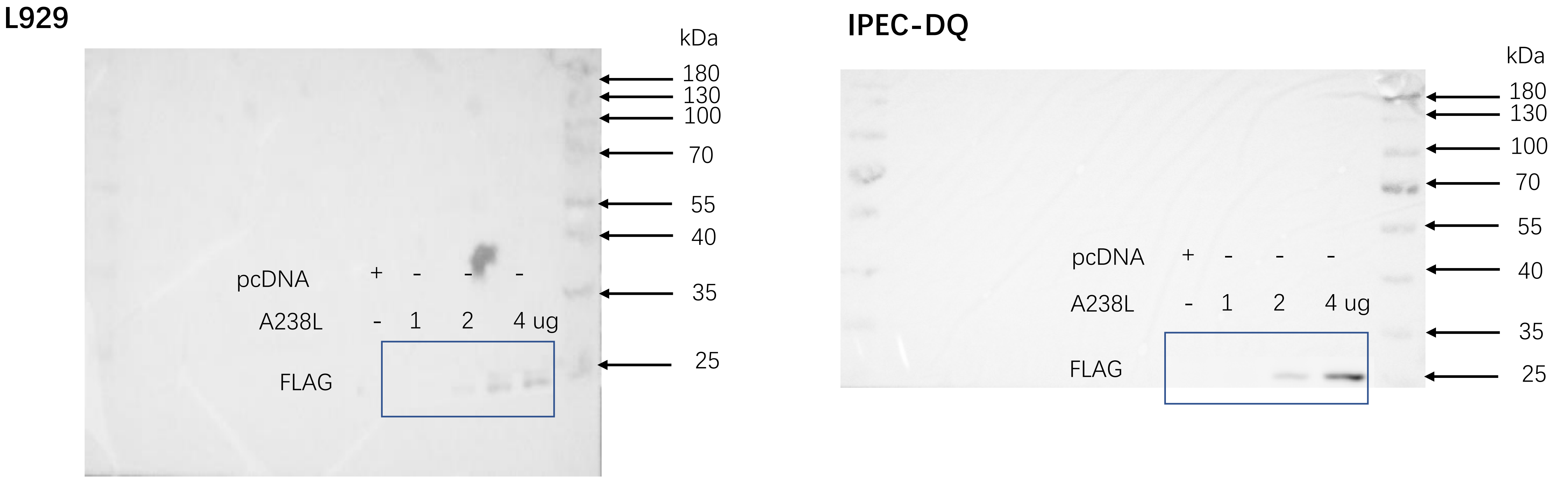

Figure S6. Western blot membrane of A238L-FLAG (~28kDa) protein detected with FLAG (DYKDDDDK Tag) (9A3) (Cat# 8146; Cell Signaling Technology) antibody. Gel-separated proteins were transferred to nitrocellulose membranes (0.2 µm pore size; Bio-Rad, Hercules, USA) by semidry electroblotting (1.5 mA per cm2, 20 min). Membranes, incubated with a horseradish peroxidase-conjugated secondary antibody (AS09 602; 1:5000–1:10000; Agrisera), were developed with Pierce™ DAB Substrate Kit (Thermo Fisher Scientific). #Weight marker (molecular weight in kDa): Vazyme Prestained Protein Ladder, 10 to 180 kDa; catalogue number: MP102-01. Blot images, prior to the densitometry readings, were converted to grayscale with ImageJ (ImageJ v.1.49, National Institutes of Health, Maryland, USA) .

3D4/21

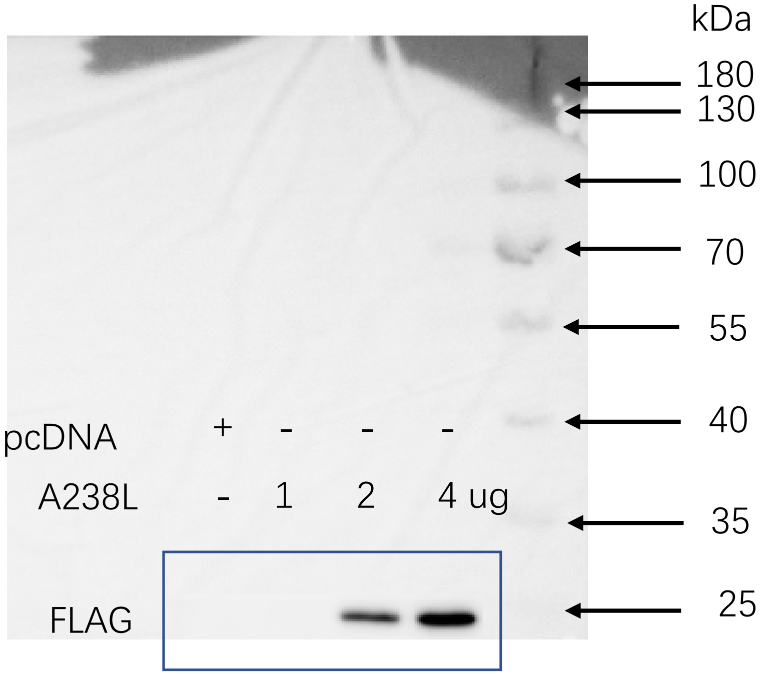

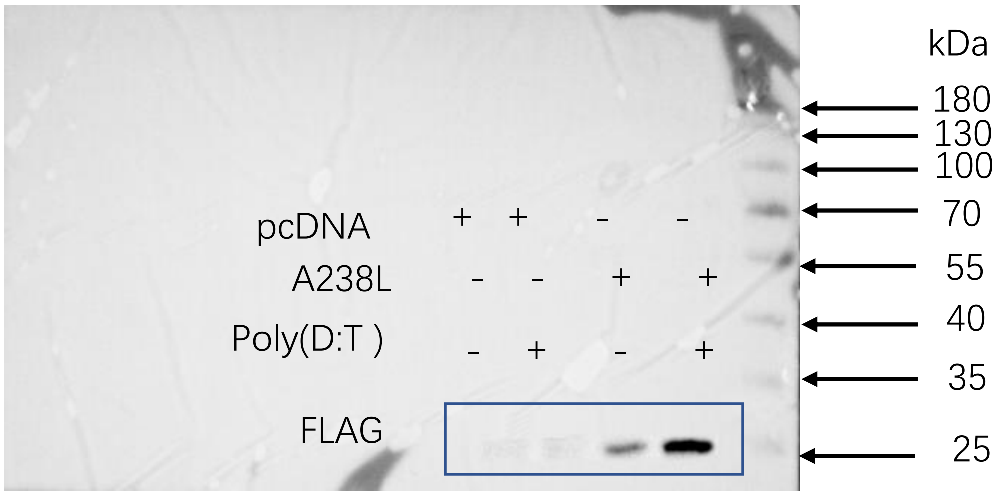

Figure S6. Western blot membrane of A238L-FLAG (~28kDa) protein detected with FLAG (DYKDDDDK Tag) (9A3) (Cat# 8146; Cell Signaling Technology) antibody. Gel-separated proteins were transferred to nitrocellulose membranes (0.2 µm pore size; Bio-Rad, Hercules, USA) by semidry electroblotting (1.5 mA per cm2, 20 min). Membranes, incubated with a horseradish peroxidase-conjugated secondary antibody (AS09 602; 1:5000–1:10000; Agrisera), were developed with Pierce™ DAB Substrate Kit (Thermo Fisher Scientific). #Weight marker (molecular weight in kDa): Vazyme Prestained Protein Ladder, 10 to 180 kDa; catalogue number: MP102-01. Blot images, prior to the densitometry readings, were converted to grayscale with ImageJ (ImageJ v.1.49, National Institutes of Health, Maryland, USA) .

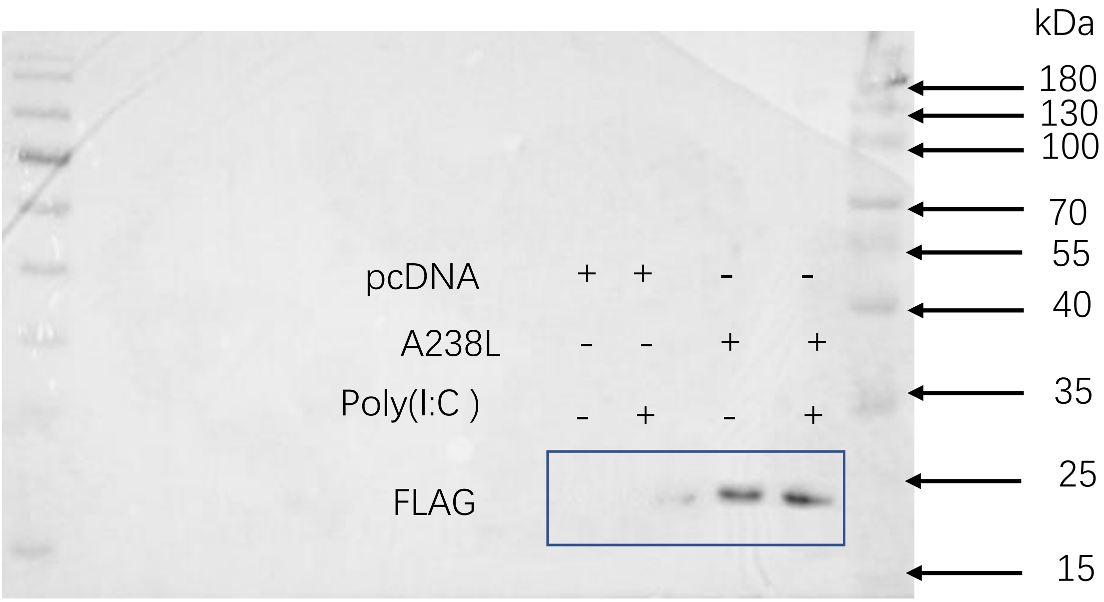

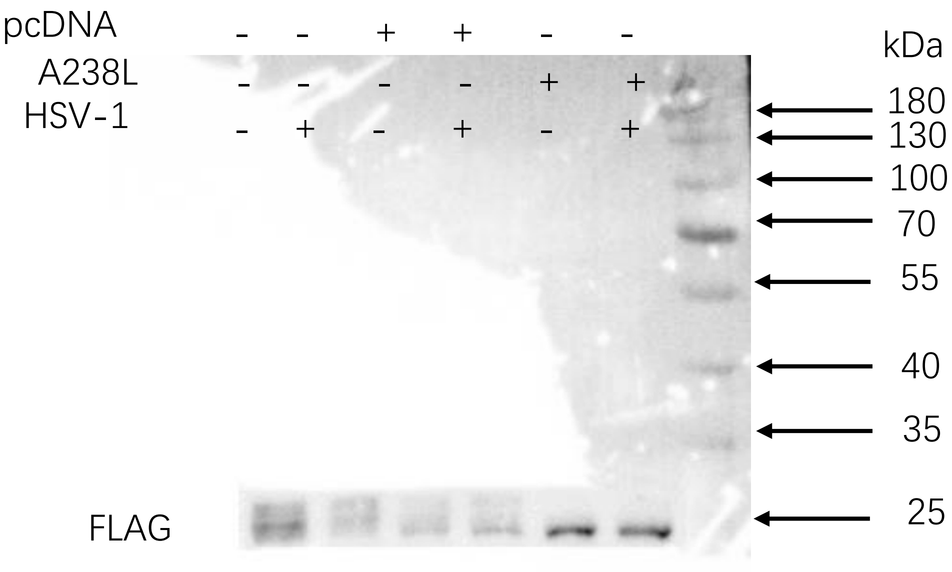

Figure S6. Western blot membrane of A238L-FLAG (~28kDa) protein detected with FLAG (DYKDDDDK Tag) (9A3) (Cat# 8146; Cell Signaling Technology) antibody. Gel-separated proteins were transferred to nitrocellulose membranes (0.2 μm pore size; Bio-Rad, Hercules, USA) by semidry electroblotting (1.5 mA per cm2, 20 min). Membranes, incubated with a horseradish peroxidase-conjugated secondary antibody (AS09 602; 1:5000–1:10000; Agrisera), were developed with Pierce™ DAB Substrate Kit (Thermo Fisher Scientific). #Weight marker (molecular weight in kDa): Vazyme Prestained Protein Ladder, 10 to 180 kDa; catalogue number: MP102-01. Blot images, prior to the densitometry readings, were converted to grayscale with ImageJ (ImageJ v.1.49, National Institutes of Health, Maryland, USA) .

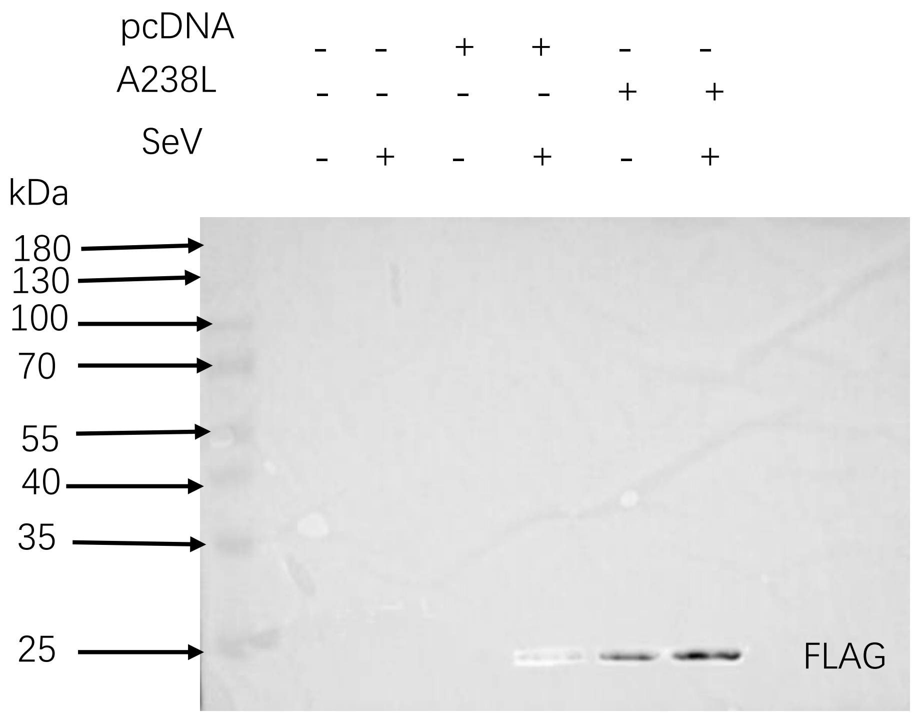

Figure S6. Western blot membrane of A238L-FLAG (~28kDa) protein detected with FLAG (DYKDDDDK Tag) (9A3) (Cat# 8146; Cell Signaling Technology) antibody. Gel-separated proteins were transferred to nitrocellulose membranes (0.2 µm pore size; Bio-Rad, Hercules, USA) by semidry electroblotting (1.5 mA per cm2, 20 min). Membranes, incubated with a horseradish peroxidase-conjugated secondary antibody (AS09 602; 1:5000–1:10000; Agrisera), were developed with Pierce™ DAB Substrate Kit (Thermo Fisher Scientific). #Weight marker (molecular weight in kDa): Vazyme Prestained Protein Ladder, 10 to 180 kDa; catalogue number: MP102-01. Blot images, prior to the densitometry readings, were converted to grayscale with ImageJ (ImageJ v.1.49, National Institutes of Health, Maryland, USA) .

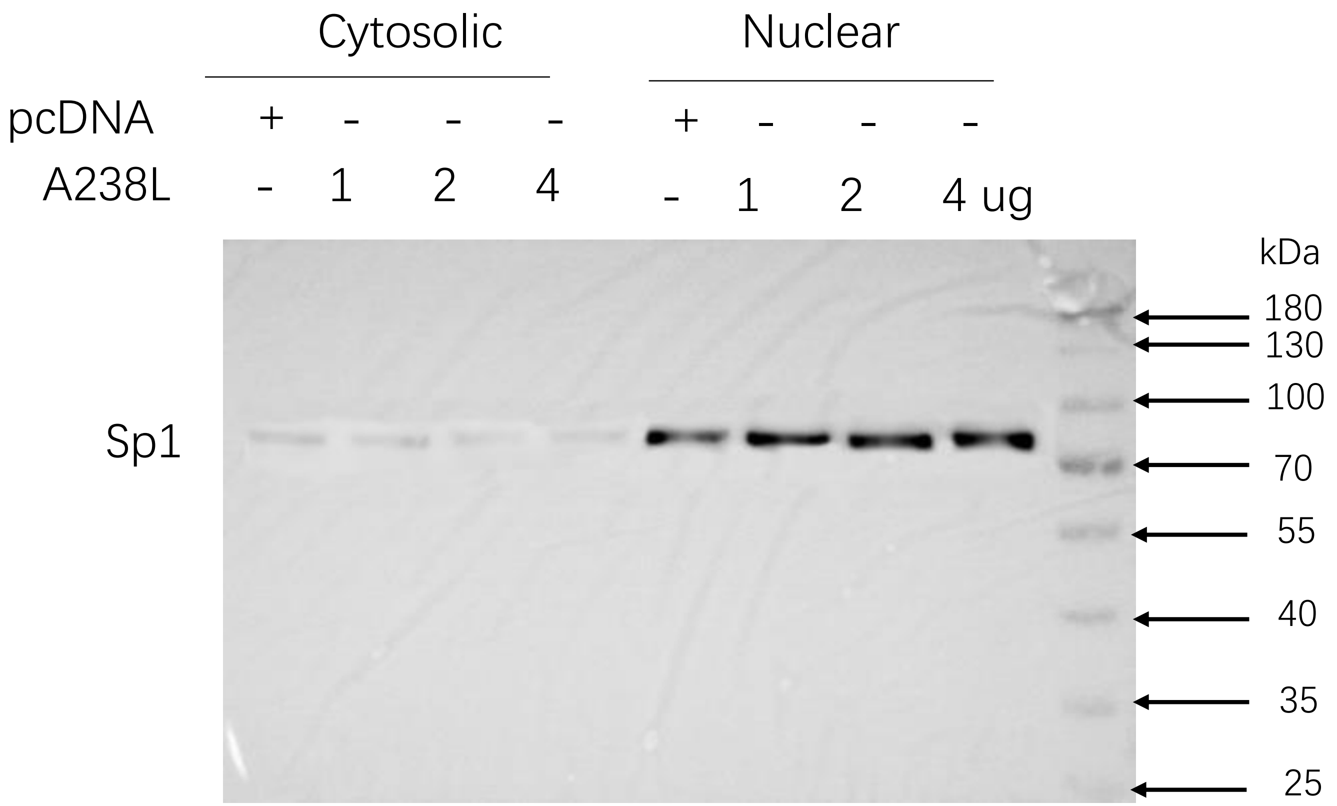

Figure S7. Western blot membrane of Sp1 (~91 kDa) protein detected with Sp1 (Cat# SC-59; 1:1000; Santa Cruz Biotechnology) antibody. Gel-separated proteins were transferred to nitrocellulose membranes (0.2 μm pore size; Bio-Rad, Hercules, USA) by semidry electroblotting (1.5 mA per cm2, 20 min). Membranes, incubated with a horseradish peroxidase-conjugated secondary antibody (AS09 602; 1:5000–1:10000; Agrisera), were developed with Pierce™ DAB Substrate Kit (Thermo Fisher Scientific). #Weight marker (molecular weight in kDa): Vazyme Prestained Protein Ladder, 10 to 180 kDa; catalogue number: MP102-01. Blot images, prior to the densitometry readings, were converted to grayscale with ImageJ (ImageJ v.1.49, National Institutes of Health, Maryland, USA) .

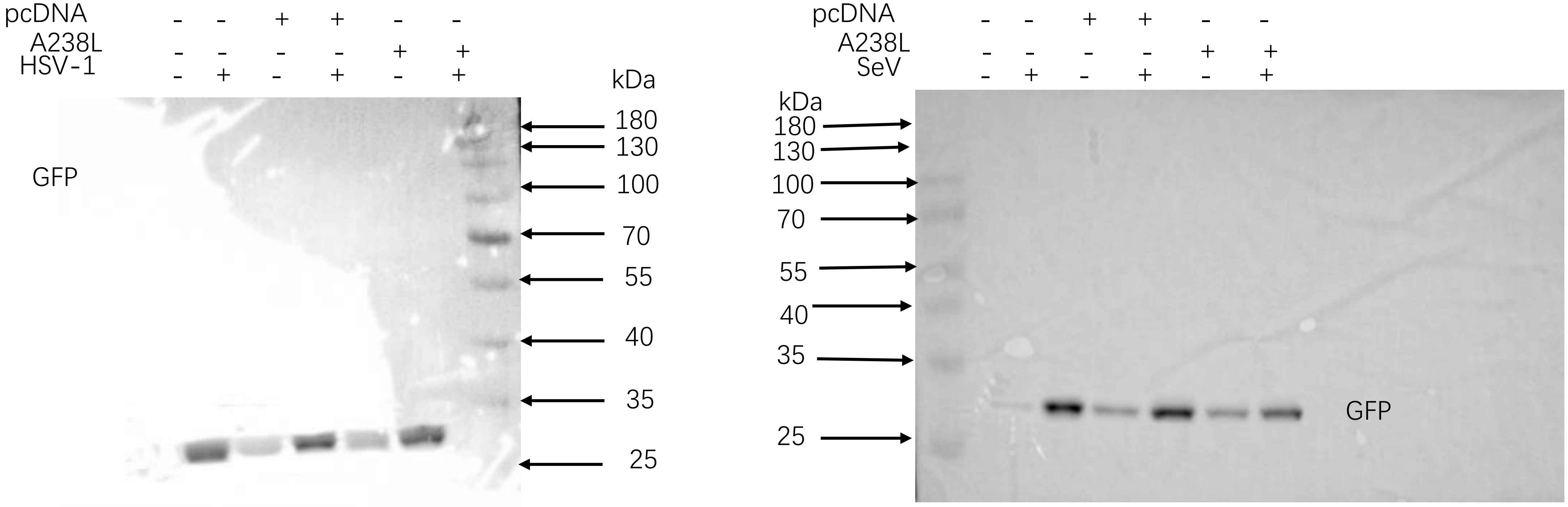

Figure S8. Western blot membrane of GFP (~27 kDa) protein detected with GFP (Cat# 2955; 1:1000; Cell Signaling Technology) antibody. Gel-separated proteins were transferred to nitrocellulose membranes (0.2 μm pore size; Bio-Rad, Hercules, USA) by semidry electroblotting (1.5 mA per cm2, 20 min). Membranes, incubated with a horseradish peroxidase-conjugated secondary antibody (AS09 602; 1:5000–1:10000; Agrisera), were developed with Pierce™ DAB Substrate Kit (Thermo Fisher Scientific). #Weight marker (molecular weight in kDa): Vazyme Prestained Protein Ladder, 10 to 180 kDa; catalogue number: MP102-01. Blot images, prior to the densitometry readings, were converted to grayscale with ImageJ (ImageJ v.1.49, National Institutes of Health, Maryland, USA) .
